# Arterial Blood Pressure and Long-Term Exposure to Traffic-Related Air Pollution: An Analysis in the European Study of Cohorts for Air Pollution Effects (ESCAPE)

**DOI:** 10.1289/ehp.1307725

**Published:** 2014-05-16

**Authors:** Kateryna B. Fuks, Gudrun Weinmayr, Maria Foraster, Julia Dratva, Regina Hampel, Danny Houthuijs, Bente Oftedal, Anna Oudin, Sviatlana Panasevich, Johanna Penell, Johan N. Sommar, Mette Sørensen, Pekka Tiittanen, Kathrin Wolf, Wei W. Xun, Inmaculada Aguilera, Xavier Basagaña, Rob Beelen, Michiel L. Bots, Bert Brunekreef, H. Bas Bueno-de-Mesquita, Barbara Caracciolo, Marta Cirach, Ulf de Faire, Audrey de Nazelle, Marloes Eeftens, Roberto Elosua, Raimund Erbel, Bertil Forsberg, Laura Fratiglioni, Jean-Michel Gaspoz, Agneta Hilding, Antti Jula, Michal Korek, Ursula Krämer, Nino Künzli, Timo Lanki, Karin Leander, Patrik K.E. Magnusson, Jaume Marrugat, Mark J. Nieuwenhuijsen, Claes-Göran Östenson, Nancy L. Pedersen, Göran Pershagen, Harish C. Phuleria, Nicole M. Probst-Hensch, Ole Raaschou-Nielsen, Emmanuel Schaffner, Tamara Schikowski, Christian Schindler, Per E. Schwarze, Anne J. Søgaard, Dorothea Sugiri, Wim J.R. Swart, Ming-Yi Tsai, Anu W. Turunen, Paolo Vineis, Annette Peters, Barbara Hoffmann

**Affiliations:** 1 IUF-Leibniz Research Institute for Environmental Medicine, Düsseldorf, Germany; 2 Institute of Epidemiology and Medical Biometry, Ulm University, Ulm, Germany; 3 Centre for Research in Environmental Epidemiology (CREAL), Barcelona, Spain; 4 CIBER Epidemiología y Salud Pública (CIBERESP), Barcelona, Spain; 5 Universitat Pompeu Fabra, Barcelona, Spain; 6 Swiss Tropical and Public Health Institute, Basel, Switzerland; 7 University of Basel, Basel, Switzerland; 8 Institute of Epidemiology II, Helmholtz Zentrum München, German Research Center for Environmental Health, Neuherberg, Germany; 9 National Institute for Public Health and the Environment (RIVM), Bilthoven, the Netherlands; 10 Division of Environmental Medicine, Norwegian Institute of Public Health, Oslo, Norway; 11 Division of Occupational and Environmental Medicine, Department of Public Health and Clinical Medicine, Umeå University, Umeå, Sweden; 12 Division of Epidemiology, Norwegian Institute of Public Health, Oslo, Norway; 13 Institute of Environmental Medicine, Karolinska Institutet, Stockholm, Sweden; 14 Danish Cancer Society Research Center, Copenhagen, Denmark; 15 Department of Environmental Health, National Institute for Health and Welfare, Kuopio, Finland; 16 Department of Epidemiology and Biostatistics, School of Public Health, Imperial College London, United Kingdom; 17 Department of Epidemiology and Public Health, University College London, London, United Kingdom; 18 Institute for Risk Assessment Sciences, Utrecht University, Utrecht, the Netherlands; 19 Julius Center for Primary Care and Health Sciences, University Medical Center Utrecht, Utrecht, the Netherlands; 20 School of Public Health, Imperial College London, London, United Kingdom; 21 Aging Research Center, Department of Neurobiology, Care Sciences and Society, Karolinska Institutet and Stockholm University, Stockholm, Sweden; 22 IMIM (Hospital del Mar Medical Research Institute), Barcelona, Spain; 23 Centre for Environmental Policy, Imperial College London, United Kingdom; 24 West German Heart Centre, University Hospital Essen, University of Duisburg-Essen, Essen, Germany; 25 Stockholm Gerontology Research Center, Stockholm, Sweden; 26 Department of Community Medicine, Primary Care and Emergency Medicine, Geneva University Hospitals, Geneva, Switzerland; 27 Faculty of Medicine, University of Geneva, Geneva, Switzerland; 28 Endocrine and Diabetes Unit, Department of Molecular Medicine and Surgery, Karolinska Institutet, Stockholm, Sweden; 29 Department of Chronic Disease Prevention, National Institute for Health and Welfare, Turku, Finland; 30 Department of Medical Epidemiology and Biostatistics, Karolinska Institutet, Stockholm, Sweden; 31 Department of Research in Inflammatory and Cardiovascular Disorders (RICAD), IMIM-Hospital del Mar, Barcelona, Spain; 32 Medical School, Heinrich Heine University of Düsseldorf, Düsseldorf, Germany

## Abstract

Background: Long-term exposure to air pollution has been hypothesized to elevate arterial blood pressure (BP). The existing evidence is scarce and country specific.

Objectives: We investigated the cross-sectional association of long-term traffic-related air pollution with BP and prevalent hypertension in European populations.

Methods: We analyzed 15 population-based cohorts, participating in the European Study of Cohorts for Air Pollution Effects (ESCAPE). We modeled residential exposure to particulate matter and nitrogen oxides with land use regression using a uniform protocol. We assessed traffic exposure with traffic indicator variables. We analyzed systolic and diastolic BP in participants medicated and nonmedicated with BP-lowering medication (BPLM) separately, adjusting for personal and area-level risk factors and environmental noise. Prevalent hypertension was defined as ≥ 140 mmHg systolic BP, or ≥ 90 mmHg diastolic BP, or intake of BPLM. We combined cohort-specific results using random-effects meta-analysis.

Results: In the main meta-analysis of 113,926 participants, traffic load on major roads within 100 m of the residence was associated with increased systolic and diastolic BP in nonmedicated participants [0.35 mmHg (95% CI: 0.02, 0.68) and 0.22 mmHg (95% CI: 0.04, 0.40) per 4,000,000 vehicles × m/day, respectively]. The estimated odds ratio (OR) for prevalent hypertension was 1.05 (95% CI: 0.99, 1.11) per 4,000,000 vehicles × m/day. Modeled air pollutants and BP were not clearly associated.

Conclusions: In this first comprehensive meta-analysis of European population-based cohorts, we observed a weak positive association of high residential traffic exposure with BP in nonmedicated participants, and an elevated OR for prevalent hypertension. The relationship of modeled air pollutants with BP was inconsistent.

Citation: Fuks KB, Weinmayr G, Foraster M, Dratva J, Hampel R, Houthuijs D, Oftedal B, Oudin A, Panasevich S, Penell J, Sommar JN, Sørensen M, Tittanen P, Wolf K, Xun WW, Aguilera I, Basagaña X, Beelen R, Bots ML, Brunekreef B, Bueno-de-Mesquita HB, Caracciolo B, Cirach M, de Faire U, de Nazelle A, Eeftens M, Elosua R, Erbel R, Forsberg B, Fratiglioni L, Gaspoz JM, Hilding A, Jula A, Korek M, Krämer U, Künzli N, Lanki T, Leander K, Magnusson PK, Marrugat J, Nieuwenhuijsen MJ, Östenson CG, Pedersen NL, Pershagen G, Phuleria HC, Probst-Hensch NM, Raaschou-Nielsen O, Schaffner E, Schikowski T, Schindler C, Schwarze PE, Søgaard AJ, Sugiri D, Swart WJ, Tsai MY, Turunen AW, Vineis P, Peters A, Hoffmann B. 2014. Arterial blood pressure and long-term exposure to traffic-related air pollution: an analysis in the European Study of Cohorts for Air Pollution Effects (ESCAPE). Environ Health Perspect 122:896–905; http://dx.doi.org/10.1289/ehp.1307725

## Introduction

Long-term exposure to traffic-related air pollution (TRAP) increases risk of cardiovascular events and mortality [Health Effects Institute (HEI) 2010]. High blood pressure (BP), a major risk factor worldwide, could mediate the cardiovascular effects of TRAP (Brook et al. 2009). It has been hypothesized that long-term exposure to TRAP could raise BP chronically and increase the risk of hypertension (Brook 2007), thereby contributing to the deleterious effects of air pollution on cardiovascular morbidity and mortality.

The evidence is very scarce so far. In two American studies with selected populations [elderly men (Schwartz et al. 2012) and black women (Coogan et al. 2012)], TRAP was linked to higher BP or hypertension. In our previous study with a German population-based cohort (Fuks et al. 2011), we found a positive association of ambient particulate matter (PM) with BP and an increased prevalence of hypertension among those living near a major road. Long-term exposure to PM and gaseous air pollutants were associated with high BP and hypertension in two large Asian cohorts (Chuang et al. 2011; Dong et al. 2013). Long-term PM concentrations were positively related to self-reported hypertension among white American adults (Johnson and Parker 2009). However, not all findings are positive. In a large population-based Danish cohort of older adults, long-term exposure to nitrogen oxides (NO_x_; indicators of TRAP), was associated with decreased BP and lower prevalence of self-reported hypertension (Sørensen et al. 2012).

In view of the sparse and partially controversial evidence, we aimed to study the effects of long-term exposure to TRAP on BP and hypertension in 15 European population-based cohorts, using a uniform methodology. We investigated the cross-sectional association of particulate air pollutants, NO_x_, and traffic indicators with arterial BP as well as with the prevalence of hypertension and intake of BP-lowering medication (BPLM). This work was performed as a part of the European Study of Cohorts for Air Pollution Effects (ESCAPE 2008).

## Methods

*General setting*. We selected existing cohort studies of mortality and chronic diseases in Europe based on their potential to quantify relationships between long-term exposure and health response. Cohorts were eligible to participate in the analysis of BP and hypertension if the following data were available: *a*) BP values, measured according to the World Health Organization (WHO) Multinational MONItoring of trends and determinants in CArdiovascular Diseases (MONICA) protocol (Hense et al. 1995) or a study-specific standard; *b*) information on BPLM use; and *c*) long-term residential TRAP concentrations at the residence, assessed with the ESCAPE land use regression (LUR) model.

A total of 15 study cohorts from nine countries were eligible to participate in this study: the national Finland Cardiovascular Risk study (FINRISK, Finland); the Danish Diet, Cancer and Health study (DCH, Denmark); the population-based Oslo Health Study (HUBRO, Norway); the Stockholm 60-year-olds cohort (60-year-olds, Sweden); the Stockholm Diabetes Preventive Program (SDPP; Sweden); the Swedish National study of Aging and Care in Kungsholmen (SNAC-K; Sweden); the Swedish Twin Registry (TwinGene); the European Prospective Investigation into Cancer and Nutrition (EPIC) cohort in Umeå, Sweden (EPIC-Umeå); the EPIC Monitoring Project on Risk Factors for Chronic Diseases (EPIC-MORGEN; the Netherlands); the EPIC Prospect cohort (EPIC-Prospect; the Netherlands); the EPIC Oxford cohort (EPIC-Oxford; the United Kingdom); the Heinz Nixdorf Risk Factors, Evaluation of Coronary Calcification, and Lifestyle (Recall) study (HNR; Germany); the Cooperative Health Research in the Region of Augsburg (KORA; Germany); the Swiss Study on Air Pollution and Lung and Heart Disease In Adults (SAPALDIA; Switzerland); and Registre Gironí del Cor–Girona’s heart registry (REGICOR; Spain). Further details on each cohort is available in Supplemental Material, “Cohort-specific information, funding and acknowledgements,” pp. 3–9. Work in all cohorts was conducted in accordance with the Declaration of Helsinki (World Medical Association 2013), and with all local ethical requirements.

*Air pollution*. Concentrations of PM, including particles with diameter ≤ 2.5 μm (PM_2.5_)_,_ ≤ 10 μm (PM_10_), > 2.5 to ≤ 10 μm (PM_coarse_; calculated as PM_10_ minus PM_2.5_), PM_2.5_ absorbance (a marker for black carbon or soot), and NO_x_ [nitrogen dioxide (NO_2_) and nitrogen monoxide (NO)] were modeled with LUR using a uniform ESCAPE procedure as described in Supplemental Material, “Land use regression model,” pp. 9–10, and elsewhere (Beelen et al. 2013; Eeftens et al. 2012). Briefly, annual averages of measured pollutant concentrations at the monitoring sites and predictor variables, derived from Europe-wide and local Geographic Information System databases were used to develop the study-specific LUR model and to predict concentrations at each participant’s address. To evaluate the impact of time-related changes in exposure, the predicted concentrations for PM_10_ and NO_2_ were back extrapolated to the time of the BP measurement using data from routine monitoring sites (see Supplemental Material, “Extrapolation of exposure values back in time,” pp. 10–11).

*Traffic indicators*. We estimated the cumulative traffic exposure with two traffic indicators, selected *a priori* by the ESCAPE consortium to ensure comparability across all study areas: *a*) total traffic load on all major roads (defined as roads with traffic intensity > 5,000 vehicles/day) within a 100-m radius buffer around the residence, defined as the sum of traffic intensity multiplied by the length of major road fragments within the buffer (vehicles times meters per day); and *b*) traffic intensity on the nearest road (any road type; vehicles per day). Both indicators were based on study area–specific road networks with traffic intensity data, based on both counted and modeled data. Time of assessment varied between study areas. We aimed to collect traffic data for different years including baseline, current, and data for years during relevant windows of exposure. For minor roads, traffic intensity data were missing in some local road networks. In these cases, missing data were imputed with a default value of 500 vehicles/day. Given that these roads were mainly minor roads, measurement error with regard to defining busy and nonbusy roads is likely small. Analyses of traffic indicator variables were adjusted for the predicted background concentration of NO_2_.

*Road traffic noise*. We took the concurrent exposure to traffic noise into account. For that, we estimated 24-hr mean road traffic noise level (*L*_den_) at the baseline address based on facade points of participants’ residences. Noise assessment was based on mandatory noise modeling according to the Directive 2002/49/EC of the European Parliament and of the Council (European Commission 2002) (see Supplemental Material, “Noise assessment,” pp. 11–12).

*Outcome assessment*. BP was measured according to the WHO standard procedure (Hense et al. 1995) in three studies (KORA, HNR, and SAPALDIA), whereas other studies applied study-specific standardized procedures (Table 1). Automated oscillometric devices (AODs) were used in nine cohorts: DCH, HUBRO, 60-year-olds, EPIC-MORGEN, EPIC-Prospect, EPIC-Oxford, HNR, SAPALDIA, and REGICOR. Three cohorts used sphygmomanometers (SDPP, SNAC-K, and EPIC-Umeå), and two cohorts used either an AOD or a sphygmomanometer (TwinGene and KORA). In most studies, BP was measured on the right arm (nine studies), in a seated position (nine studies), and using different cuff sizes according to the upper arm circumference (all except FINRISK). BP was measured at least twice, with a minimum pause of 2 min, in all cohorts but SDPP and a part of EPIC-Oxford. In DCH, if the first measured BP value was considered abnormal, a new measurement was taken 3 min later. The lowest BP measurement was recorded as final.

**Table 1:** BP measurement procedure in the participating cohorts.

Study	Measurement period	WHO protocol^*a*^	Arm used	Different cuff sizes	Body position	Measurement device	Repeated measurements	Final BP
FINRISK	1992, 1997, 2002, 2007	No	Right	No	Sitting	Manual mercury SM	2–3^*b*^	Mean (1st–2nd)
DCH	1993–1997	No	Right	Yes	Supine	AOD	1–2^*c*^	1st
HUBRO	2000–2001	No	Right	Yes	Sitting	AOD	3	Mean (2nd–3rd)
60-year-olds	1997–1999	No	Right	Yes	Supine	AOD	2	Mean (1st–2nd)
SDPP	1992–1994, 1996–1998	No	Either	Yes	Sitting	Manual SM	1	1st
SNAC-K	2001–2004	No	Left	Yes	Sitting, supine, standing	Manual SM	4	2nd
TwinGene	2004–2008	No	Right	Yes	Sitting	AOD, manual SM	2	Mean
EPIC-Umeå	1992–1996	No	Right	Yes	Sitting, supine	Manual SM	2	Mean
EPIC-MORGEN	1993–1997	No	Left	Yes	Supine	AOD	2	Mean
EPIC-Prospect	1993–1997	No	Left	No	Supine	AOD	2	Mean
EPIC-Oxford	1993–2001	No	Either	Yes	Sitting	AOD	1–2^*d*^	Last
HNR	2000–2003	Yes	Right	Yes	Sitting	AOD^*e*^	3	Mean (2nd–3rd)
KORA	1994–1995, 1999–2001	Yes	Right	Yes	Sitting	Random-zero SM, AOD	3	Last
SAPALDIA	2001–2002	Yes	Left	Yes	Sitting	AOD	2	Mean
REGICOR	2003–2006	No	Right	Yes	Sitting	AOD	2^*f*^	Last
SM, sphygmomanometer. ^***a***^Hense et al. (1995). ^***b***^Two BP measurements were performed in 1992, 1997; three measurements in 2002, 2007. ^***c***^If the first measured BP value was considered abnormal, a new measurement was taken 3 min later; the lowest BP measurement was recorded as final. ^***d***^BP was measured twice in a subset of 5,241 participants. ^***e***^The missing BP value with AOD was replaced with the value recorded with random-zero SM (in 34 participants, 0.7% of the sample). ^***f***^If the difference between the first and the second measurement was > 5 mmHg, a third measurement was performed.								

Intake of BPLM at baseline was assessed by questionnaire or interview and was available in 14 studies. Twelve cohorts had detailed information on the name of the drug, whereas two cohorts only had self-reported information on intake of any BPLM (see Supplemental Material, “Assessment of blood pressure lowering medication use,” p. 12). Hypertension was defined as systolic BP ≥ 140 mmHg, diastolic BP ≥ 90 mmHg, or current intake of BPLM (Chobanian et al. 2003). Intake of BPLM was examined as an additional outcome.

*Statistical analyses in cohorts*. We conducted the analyses in each cohort separately; no pooling of individual data was done. Cohort-specific analyses were performed in each study center according to a uniform statistical protocol, which is briefly described below (for more details, see Supplemental Material, “Cohort-specific analysis,” pp. 12–13). We used STATA versions 10–12 (StataCorp; http://www.stata.com). BP readings were treated as continuous outcomes; hypertension and intake of BPLM, as dichotomous outcomes. Analyses of systolic and diastolic BP were performed with linear regression. For analyses of BPLM intake and hypertension, logistic regression was used. Linear regression model fit and assumptions were tested in each cohort (see Supplemental Material, “Cohort-specific analysis,” pp. 12–13). Results were presented for the fixed increments of exposures, harmonized across all ESCAPE publications (see Supplemental Material, “Exposure increments in the analyses,” p. 11).

*Correcting for the effect of antihypertensive medication*. To account for the influence of BPLM intake on the level of measured BP, we assessed the effect of air pollution on BP in participants taking BPLM (“medicated”) and in participants not taking BPLM (“nonmedicated”) separately. To increase power, we calculated results in subgroups of medicated and nonmedicated in the whole cohort, using an interaction term, exposure × BPLM intake. The analysis model was

*BP =* β_0_ *+* β_1_ *×* Exposure *+* β_2_ *× BPLM +* β_3_ *×* Exposure *× BPLM +…+* β*_k_ ×* Covariate*_k_ +* ε. [1]

BPLM intake was coded as 0 (no medication) or 1 (medication). The effect of exposure on BP in medicated (*BPLM* = 1) participants was therefore estimated as

β_1_ *×* Exposure *+* β_3_ × Exposure *×* 1 *=* (β_1_ *+* β_3_) *×* Exposure. [2]

In nonmedicated participants (*BPLM* = 0),

β_1_ *×* Exposure *+* β_3_ *×* Exposure *×* 0 *=* β_1_ *×* Exposure. [3]

We used the *Z*-test for interaction with pooled (meta-analysis) estimates in medicated and nonmedicated.

We also conducted a sensitivity analysis with normal right-censored regression to account for BPLM effect. With this method, BP in medicated participants was censored as right-censored (Tobin et al. 2005). The normal censored regression is fit in Equation 1, assuming that the underlying BP in the medicated participants is equal or higher than the measured value under medication:

*BP*_underlying_ ≥ *BP*_measured_ if *BPLM =* 1 *BP*_underlying_ = *BP*_measured_ if *BPLM =* 0. [4]

*Covariates included in the analysis*. We used harmonized definitions of covariates and adjustment sets. The adjustment sets were defined *a priori* using causal graphs (Glymour and Greenland 2008). The main model included age (years), sex (male, female), body mass index (BMI; kilograms per meter squared), smoking status (smoker, ex-smoker, nonsmoker), pack-years of smoking (total pack-years smoked), passive smoking (yes, no), alcohol consumption (never, 1–3 drinks/week, 3–6 drinks/week, > 6 drinks/week; if wine was assessed separately, alcohol consumption excluding wine was calculated), wine consumption (drinks per week; if available), physical activity (< once per month or < 1 hr/week, once per week or 1 hr/week, 2–3 times/week or > 1 and < 3 hr/week, > 3 times/week or > 3 hr/week), individual socioeconomic status [SES; defined as educational level (≤ primary school, ≤ secondary school or equivalent, ≥ university degree)] and economic activity (employed/self-employed, unemployed, homemaker/housewife, retired).

In cases where a covariate was not available, was of low quality, or contained > 10% missing values, the covariate was replaced by a similar covariate or excluded from the individual cohort-specific model. For example, instead of physical activity in categories (which was not available in REGICOR), a weekly leisure time physical activity variable was used.

Based on existing knowledge of possible nonlinear relationships for age, BMI, pack-years of smoking, and wine consumption (where available), the corresponding terms were entered as linear and squared, centered on the mean.

*Controlling for area-level effects*. To adjust for potential clustering of the outcome on a small-scale spatial level, we included a random intercept for neighborhood in the mixed-effects regression models. If area-level variables were available at different spatial scales, we used the scale corresponding to the spatial scale of the random intercept, which was chosen based on the Akaike information criterion of the model. In addition, we controlled for potential confounding on the area level by including the information on neighborhood SES as a covariate in the main model. If available, we used unemployment rate in the neighborhood, or, alternatively, welfare rate, average education level, or mean income.

*Meta-analysis*. The random effects meta-analysis based on the DerSimonian and Laird (1986) method was performed. We defined the *p*-value of Cochrane’s *Q*-test < 0.05 or an *I*^2^ > 50% as an indication for heterogeneity (Higgins and Thompson 2002). Forest plots were produced using the package metafor (Viechtbauer 2010) in R version 2.13.1 (R Project for Statistical Computing; http://www.r-project.org/).

As sensitivity analyses, we divided cohorts into groups by quality of BP measurement procedure and excluded studies one by one to investigate the impact of individual studies on the meta-estimate. We also conducted meta-regression using characteristics of population and exposure in the cohort as independent predictors. For further details, see Supplemental Material, “Sensitivity meta-analysis and meta-regression,” pp. 13–15.

## Results

We analyzed data from 15 cohorts in nine European countries, comprising 164,484 individuals with information on exposure, outcome, and covariates (Table 2). Cohort-specific baseline examinations were performed over a period that ranged from 1992 until 2008. Two cohorts were excluded from the main meta-analysis: EPIC-Oxford, due to information on BPLM not being available, and DCH, due to a slightly different BP measurement method in hypertensive participants (“Methods”; see also Supplemental Material, Table S1). This left 13 cohorts with 113,926 participants in the main meta-analysis of NO_x_ and traffic load, and 12 cohorts with 90,852 participants in the main analysis of PM. All 15 cohorts were included in the extended meta-analysis.

**Table 2:** Description of the study population in the cohorts included in the main and the extended meta-analysis.

Study (country)	Participants (*n*)	Systolic BP (mean ± SD)	Diastolic BP (mean ± SD)	BPLM (%)	Hypertension (%)	Age (mean ± SD)	Men (%)	BMI [kg/m^2 ^(mean ± SD)]	Smokers (%)
FINRISK (Finland)	10,318	134.1 ± 19.3	80.7 ± 11.6	12.7	41.6	48.1 ± 13.2	47.0	26.4 ± 4.6	26.7
HUBRO (Norway)	16,200	130.3 ± 17.8	75.0 ± 11.2	11.8	32.0	47.8 ± 15.1	44.7	25.6 ± 4.1	25.4
60-year-olds (Sweden)	3,659	138.4 ± 21.8	84.5 ± 10.6	19.6	52.7	60.4 ± 0.1	47.1	26.8 ± 4.2	19.9
SDPP (Sweden)	7,535	122.8 ± 15.9	77.0 ± 10.0	5.8	24.0	47.1 ± 4.9	38.5	25.7 ± 4.0	26.1
SNAC-K (Sweden)	2,738	142.7 ± 20.2	81.3 ± 10.6	9.8	66.3	71.1 ± 9.5	41.7	25.7 ± 3.9	13.6
TwinGene (Sweden)	1,296	135.6 ± 18.8	83.8 ± 11.5	21.4	55.5	60.9 ± 6.0	39.7	25.2 ± 3.7	20.2
EPIC-Umeå (Sweden)	21,912	126.7 ± 17.2	78.6 ± 10.6	7.5	34.8	46.0 ± 10.2	47.8	25.0 ± 4.0	18.9
EPIC-MORGEN (Netherlands)	16,293	120.8 ± 16.3	76.8 ± 10.7	22.9	20.5	43.9 ± 10.9	45.2	25.2 ± 4.0	34.4
EPIC-Prospect (Netherlands)	16,434	132.5 ± 20.5	78.8 ± 10.8	20.4	43.4	57.7 ± 6.0	0	25.5 ± 4.1	22.2
HNR (Germany)	4,615	133.1 ± 20.8	81.4 ± 10.9	35.3	56.9	59.5 ± 7.8	49.9	27.9 ± 4.6	23.2
KORA (Germany)	7,501	131.0 ± 19.6	80.7 ± 10.9	18.5	41.0	50.5 ± 13.6	49.0	27.3 ± 4.6	24.4
SAPALDIA (Switzerland)	1,884^*a*^	126.1 ± 18.3	80.3 ± 10.5	19.3	37.3	53.3 ± 11.4	46.5	25.4 ± 4.2	27.1
REGICOR (Spain)	3,541	127.7 ± 19.9	78.4 ± 10.2	25.8	41.7	57.7 ± 12.3	45.2	27.0 ± 4.4	19.8
TOTAL_main_	113,926^*b*^	130.9	79.8	13.1	36.0	54.1	38.8	26.0	24.2
DCH (Denmark)	36,829	140.4 ± 20.6	83.4 ± 10.6	13.0	55.19	56.8 ± 4.4	47.1	26.0 ± 4.1	37.0
EPIC-Oxford (UK)	13,729	126.0 ± 19.1	77.1 ± 11.1	—	32.4	49.6 ± 11.6	22.8	24.5 ± 4.1	—
TOTAL_extended_	164,484	131.2	79.8	12.0	40.0	54.0	39.3	25.9	25.0
Studies in the main meta-analysis are ordered from north to south. ^***a***^Data on NO_x_ and traffic indicators were available for all three sites of SAPALDIA: Basel, Geneva, Lugano (*n* = 1,884). PM exposure concentrations were available only for the Lugano site (*n* = 722). ^***b***^*n* = 90,852 in the analysis of PM exposures. PM was not modeled in EPIC-Umeå and in two of three sites of SAPALDIA.									

Of the 113,926 participants in the main meta-analysis with NO_x_ and traffic load in a 100-m buffer, 14,943 participants (13.1%) were taking BPLM and 41,067 (36.0%) had hypertension. Mean systolic BP in cohorts ranged from 120.8 mmHg to 142.7 mmHg; mean diastolic BP ranged from 75.0 mmHg to 84.5 mmHg (Table 2). Characteristics of participants included in the main analysis were similar to the extended sample (Table 2).

Mean pollutant concentrations increased from north to south across the studies (Table 3). Correlation between pollutant concentrations ranged from moderate (Pearson’s ρ = 0.5–0.7) to high (ρ > 0.7) (see Supplemental Material, Table S2). We observed a high correlation of PM measures, of PM with NO_x_, and of NO_2_ with NO_x_ in most study areas. We observed moderate-to-high correlations between pollutants, traffic indicators, and road traffic noise. The two traffic indicators were weakly (ρ = 0.3–0.5) to moderately correlated.

**Table 3:** Characteristics of the LUR model (leave-one-out cross-validation *R^2^*) and concentrations of long-term TRAP in cohorts (mean ± SD).

Study	*R*^2^ LUR validation		PM_2.5_ (μg/m^3^)	PM_2.5_ absorbance (10^–5^/m)	PM_coarse _(μg/m^3^)	PM_10_ (μg/m^3^)	NO_2_ (μg/m^3^)	NO_x_ (μg/m^3^)	Traffic load (10^6^ vehicles × m/day)
	PM_2.5_ (%)^*a*^	NO_2_ (%)^*b*^							
FINRISK	53	75	7.7 ± 1.1	0.9 ± 0.2	6.6 ± 2.3	14.0 ± 3.1	15.3 ± 4.9	24.2 ± 8.8	0.6 ± 1.5
HUBRO	68	66	9.0 ± 1.3	1.2 ± 0.3	4.0 ± 2.0	13.5 ± 3.1	20.9 ± 7.9	38.3 ± 15.3	0.8 ± 1.9
60-year-olds	78^*c*^	83	7.3 ± 1.3	0.6 ± 0.2	7.4 ± 2.9	15.0 ± 3.8	10.8 ± 4.2	10.3 ± 3.6	0.5 ± 1.5
SDPP	78^*c*^	83	6.6 ± 1.2	0.5 ± 0.1	6.3 ± 2.4	13.7 ± 3.2	8.4 ± 1.7	14.4 ± 3.3	0.1 ± 0.4
SNAC-K	78^*c*^	83	7.9 ± 1.3	0.8 ± 0.2	8.5 ± 4.7	16.3 ± 6.0	17.4 ± 4.8	33.1 ± 12.3	2.2 ± 3.7
TwinGene	78^*c*^	83	7.3 ± 1.3	0.6 ± 0.2	7.2 ± 3.0	14.8 ± 4.0	10.7 ± 4.0	18.4 ± 8.9	0.6 ± 1.7
EPIC-Umeå	—	83	—	—	—	—	5.2 ± 2.4	8.7 ± 5.7	0.1 ± 0.4
EPIC-MORGEN	61	81	16.9 ± 0.6	1.4 ± 0.2	8.6 ± 1.1	25.4 ± 1.7	23.8 ± 7.0	36.4 ± 11.7	0.9 ± 2.0
EPIC-Prospect	61	81	16.8 ± 0.5	1.4 ± 0.2	8.5 ± 0.7	25.3 ± 1.2	26.7 ± 4.7	39.6 ± 10.6	0.7 ± 1.6
HNR	79	84	18.4 ± 1.1	1.6 ± 0.4	10.0 ± 1.8	27.8 ± 1.9	30.2 ± 4.9	50.8 ± 12.0	1.0 ± 2.2
KORA	62	67	13.6 ± 0.9	1.7 ± 0.2	6.2 ± 1.1	20.3 ± 2.4	18.7 ± 3.9	32.6 ± 7.4	0.4 ± 1.1
SAPALDIA	77^*d*^	58^*d*^, 82^*e*^	17.1 ± 1.4^*d*^	2.0 ± 0.4^*d*^	6.7 ± 1.2^*d*^	23.7 ± 2.2^*d*^	27.5 ± 6.4^*f*^	46.0 ± 13.8^*f*^	1.0 ± 1.8^*f*^
REGICOR	71	68	15.0 ± 1.7	2.3 ± 0.7	15.0 ± 2.4	32.0 ± 4.0	35.5 ± 14.2	63.2 ± 29.1	1.6 ± 2.3
TOTAL (main)			12.0	1.2	7.9	20.2	19.3	32.0	0.8
DCH	55	83	11.3 ± 0.9	1.15 ± 0.2	5.7 ± 1.0	17.1 ± 1.9	16.3 ± 7.0	26.6 ± 18.3	1.2 ± 2.3
EPIC-Oxford	77	87	9.7 ± 1.0	1.05 ± 0.2	6.4 ± 0.9	16.0 ± 2.0	22.9 ± 7.2	38.3 ± 14.0	0.4 ± 1.3
TOTAL (extended)			11.7	1.2	7.6	19.6	19.4	32.1	0.8
^***a***^Eeftens et al. (2012). ^***b***^Beelen et al. (2013). ^***c***^Common model was developed for the Stockholm cohorts: 60-year-olds, SDPP, SNAC-K, TwinGene. ^***d***^Only Lugano site of SAPALDIA. ^***e***^Only Basel and Geneva sites of SAPALDIA. ^***f***^Three sites of SAPALDIA (Basel, Geneva, Lugano).									

**Figure 1:**
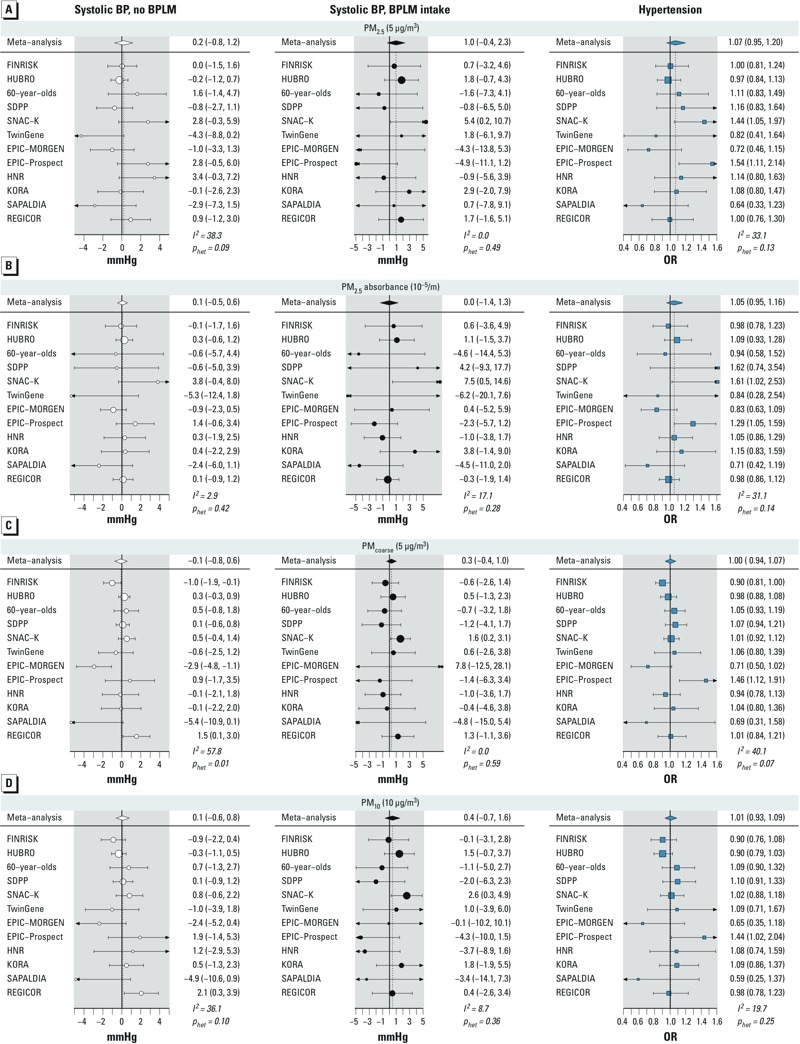
Cohort-specific and meta-analysis estimates of association of PM_2.5_ (*A*)_,_ absorbance PM_2.5_ (*B*), PM_coarse_ (*C*), and PM_10_ (*D*) with systolic BP and hypertension. Results are presented per given increments. *I*^2^ is a measure of heterogeneity between cohorts, and *p_het_* is a *p*-value for the *Q*-test of heterogeneity.

*Associations with particulate air pollutants*. Modeled PM concentrations were not clearly associated with any of the studied outcomes in the single-pollutant models (Tables 4, 5 and Figure 1). We found a 0.20-mmHg (95% CI: –0.76, 1.16) and a 0.98-mmHg (95% CI: –0.35, 2.31) increase in systolic BP per 5-μg/m^3^ increase in PM_2.5_ in nonmedicated and medicated participants, respectively. The *p*_interaction_ for PM_2.5_ × BPLM intake was 0.25. Similar results were found for diastolic BP: an increase of 0.14 mmHg in nonmedicated (95% CI: –0.57, 0.85) and by 0.59 mmHg in medicated (95% CI: –0.19, 1.37) participants per 5-μg/m^3^ increase in PM_2.5_; the *p*_interaction_ was 0.26. The ORs for hypertension and BPLM intake per 5-μg/m^3^ of PM_2.5_ were 1.07 (95% CI: 0.95, 1.21) and 1.06 (95% CI: 0.96, 1.17), respectively. Similarly, elevated, but nonsignificant, estimates were observed for PM_2.5_ absorbance, PM_coarse_, and PM_10_. Results across studies were somewhat heterogeneous for PM_2.5_ and PM_coarse_ (Figure 1), displaying relatively large positive point estimates in some cohorts and inverse associations in others.

**Table 4:** Adjusted^*a*^ associations of TRAP and traffic indicators with BP, estimated with random-effects meta-analysis.

Outcome and exposure (increment)	Studies (*n*)	No BPLM	*p*_*het*_	*I*^2^ (%)	BPLM intake	*p*_*het*_	*I*^2^ (%)
		Change^*b*^ [mmHg (95% CI)]			Change [mmHg (95% CI)]		
Systolic BP
PM_2.5_ (5 μg/m^3^)	12^*c*^	0.20 (–0.76, 1.16)	0.09	38	0.98 (–0.35, 2.31)	0.49	0
PM_2.5_ absorbance (10^–5^/m)	12	0.07 (–0.46, 0.60)	0.42	3	–0.04 (–1.37, 1.29)	0.28	17
PM_coarse_ (5 μg/m^3^)	12	–0.09 (–0.76, 0.58)	0.01	58	0.30 (–0.44, 1.04)	0.59	0
PM_10_ (10 μg/m^3^)	12	0.09 (–0.60, 0.78)	0.10	36	0.44 (–0.68, 1.56)	0.36	9
NO_2_ (10 μg/m^3^)	13	–0.29 (–0.70, 0.12)	0.02	50	–0.14 (–0.77, 0.49)	0.26	18
NO_x_ (20 μg/m^3^)	13	–0.08 (–0.47, 0.31)	0.03	48	0.04 (–0.43, 0.51)	0.61	0
Traffic load (4 × 10^6^ vehicles × m/day)	13^*d*^	0.35 (0.02, 0.68)	0.35	9	–0.11 (–0.74, 0.52)	0.84	0
Traffic intensity (5,000 vehicles/day)	12^*e*^	0.08 (–0.06, 0.22)	0.86	0	0.11 (–0.22, 0.45)	0.73	0
Diastolic BP
PM_2.5_ (5 μg/m^3^)	12^*c*^	0.14 (–0.57, 0.85)	0.01	57	0.59 (–0.19, 1.37)	0.88	0
PM_2.5_ absorbance (10^–5^/m)	12	0.24 (–0.09, 0.57)	0.4	5	0.43 (–0.49, 1.35)	0.14	32
PM_coarse_ (5 μg/m^3^)	12	0.13 (–0.11, 0.37)	0.25	20	0.34 (–0.23, 0.91)	0.13	32
PM_10_ (10 μg/m^3^)	12	0.17 (–0.12, 0.46)	0.31	14	0.63 (–0.11, 1.37)	0.23	22
NO_2_ (10 μg/m^3^)	13	0.04 (–0.10, 0.18)	0.62	0	0.21 (–0.12, 0.54)	0.32	13
NO_x_ (20 μg/m^3^)	13	0.09 (–0.05, 0.23)	0.62	0	0.32 (–0.01, 0.65)	0.30	14
Traffic load (4 × 10^6^ vehicles × m/day)	13^*d*^	0.22 (0.04, 0.40)	0.72	0	–0.04 (–0.39, 0.31)	0.94	0
Traffic intensity (5,000 vehicles/day)	12^*e*^	0.08 (0.00, 0.16)	0.80	0	–0.04 (–0.30, 0.21)	0.22	22
*I*^2^ is a measure of heterogeneity between cohorts, and *p*_het_ is a *p*-value for the *Q*-test of ­heterogeneity. ^***a***^Adjusted for age, sex, BMI, smoking status, pack-years of smoking, passive smoking, alcohol consumption, physical activity, educational level, economic activity, neighborhood SES (including a random intercept for a neighborhood). ^***b***^Estimated change in BP refers to the indicated exposure increment. ^***c***^FINRISK, HUBRO, 60-year-olds, SDPP, SNAC-K, TwinGene, EPIC-MORGEN, EPIC-Prospect, HNR, KORA, SAPALDIA (Lugano site), REGICOR; *n*(total) = 91,574; *n*(non­medicated) = 79,404; *n*(medicated) = 12,170. ^***d***^FINRISK, HUBRO, 60-year-olds, SDPP, SNAC-K, TwinGene, EPIC-Umeå, EPIC-MORGEN, EPIC-Prospect, HNR, KORA, SAPALDIA, REGICOR; *n*(total) = 114,648; *n*(non­medicated) = 99,705; *n*(medicated) = 14,943. ^***e***^FINRISK, HUBRO, 60-year-olds, SDPP, SNAC-K, TwinGene, EPIC-Umeå, EPIC-MORGEN, EPIC-Prospect, KORA, SAPALDIA, REGICOR; *n*(total) = 110,033; *n*(non­medicated) = 96,717; *n*(medicated) = 13,316.							

**Table 5:** Adjusted^*a*^ associations of TRAP and traffic indicators with prevalent hypertension and BPLM intake as outcomes, estimated with random-effects meta-analysis.

Outcome and exposure (increment)	Studies (*n*)	OR^*b*^ (95% CI)	*p*_*het*_	*I*^2^
Hypertension as outcome
PM_2.5_ (5 μg/m^3^)	12^*c*^	1.07 (0.95, 1.21)	0.13	33
PM_2.5_ absorbance (10^–5^/m)	12	1.05 (0.95, 1.16)	0.14	31
PM_coarse_ (5 μg/m^3^)	12	1.00 (0.94, 1.06)	0.07	40
PM_10_ (10 μg/m^3^)	12	1.01 (0.93, 1.09)	0.25	20
NO_2_ (10 μg/m^3^)	13	0.98 (0.92, 1.04)	0.01	55
NO_x_ (20 μg/m^3^)	13	0.98 (0.92, 1.04)	< 0.01	64
Traffic load (4 × 10^6^ vehicles × m/day)	13^*d*^	1.05 (0.99, 1.11)	0.02	51
Traffic intensity (5,000 vehicles/day)	12^*e*^	1.02 (1.00, 1.04)	0.38	7
BPLM intake as outcome
PM_2.5_ (5 μg/m^3^)	12^*c*^	1.06 (0.96, 1.17)	0.85	0
PM_2.5_ absorbance (10^–5^/m)	12	1.08 (0.98, 1.19)	0.24	20
PM_coarse_ (5 μg/m^3^)	12	0.99 (0.93, 1.05)	0.63	0
PM_10_ (10 μg/m^3^)	12	0.98 (0.91, 1.06)	0.54	0
NO_2_ (10 μg/m^3^)	13	1.01 (0.97, 1.05)	0.30	14
NO_x_ (20 μg/m^3^)	13	0.98 (0.94, 1.02)	0.60	0
Traffic load (4 × 10^6^ vehicles × m/day)	13^*d*^	1.04 (0.98, 1.10)	0.12	33
Traffic intensity (5,000 vehicles/day)	12^*e*^	1.00 (0.98, 1.02)	0.76	0
*I*^2^ is a measure of heterogeneity between cohorts, and *p*_het_ is a *p*-value for the *Q*-test of heterogeneity. ^***a***^Adjusted for age, sex, BMI, smoking status, pack-years of smoking, passive smoking, alcohol consumption, physical activity, educational level, economic activity, neighborhood SES (including a random intercept for a neighborhood). ^***b***^OR for the indicated exposure increment. ^***c***^FINRISK, HUBRO, 60-year-olds, SDPP, SNAC-K, TwinGene, EPIC-MORGEN, EPIC-Prospect, HNR, KORA, SAPALDIA (Lugano site), REGICOR; *n* = 91,574. ^***d***^FINRISK, HUBRO, 60-year-olds, SDPP, SNAC-K, TwinGene, EPIC-Umeå, EPIC-MORGEN, EPIC-Prospect, HNR, KORA, SAPALDIA, REGICOR; *n* = 114,648. ^***e***^FINRISK, HUBRO, 60-year-olds, SDPP, SNAC-K, TwinGene, EPIC-Umeå, EPIC-MORGEN, EPIC-Prospect, KORA, SAPALDIA, REGICOR; *n* = 110,033.				

**Figure 2:**
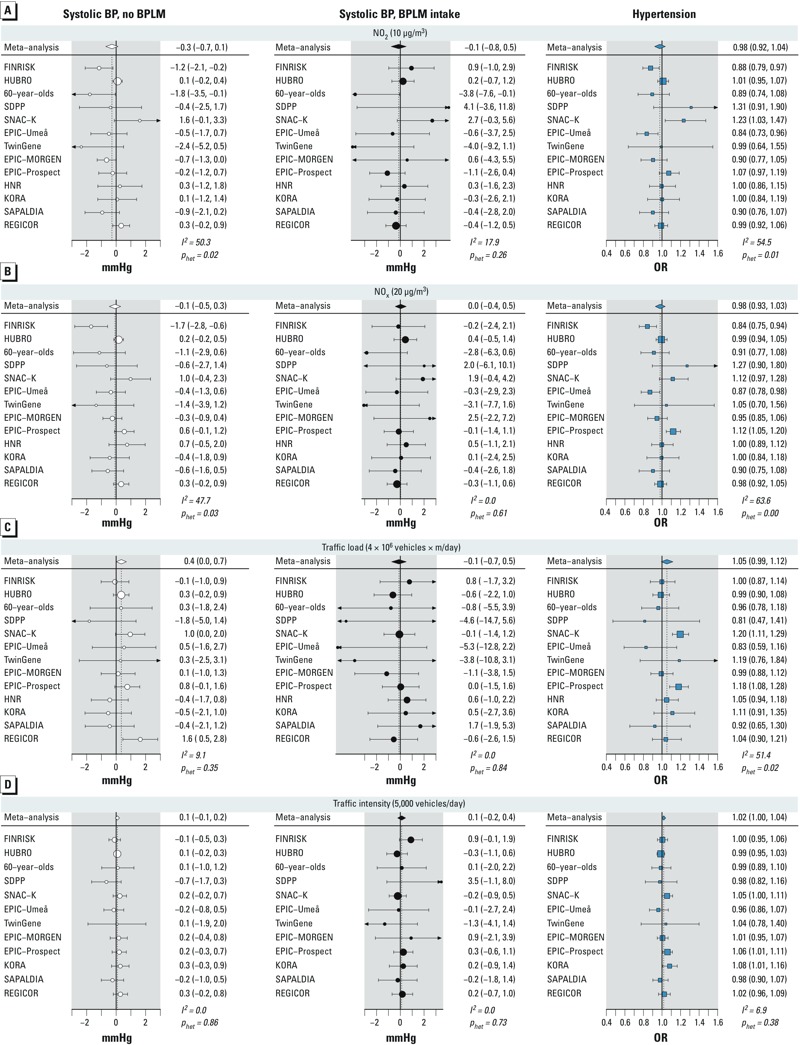
Cohort-specific and meta-analysis estimates of association of NO_2_ (*A*), NO_x_ (*B*), traffic load at major road fragments (*C*), and traffic intensity at the nearest road (*D*) with systolic BP and hypertension. Results are presented per given increments. *I*^2^ is a measure of heterogeneity between cohorts, and *p_het_* is a *p*-value for the *Q*-test of heterogeneity.

*Associations with NO_x_*. Modeled concentrations of NO_x_ were not significantly associated with any of the outcomes, although NO_2_ showed a weak inverse relationship with systolic BP in nonmedicated participants (–0.29; 95% CI: –0.70, 0.12) mmHg per 10-μg/m^3^; the *p*_interaction_ with BPLM intake was 0.64). Results were similar for NO_x_ (Tables 4, 5 and Figure 2). Significant heterogeneity was observed in the meta-analysis of NO_2_ and NO_x_ with BP in nonmedicated participants and in the analysis with hypertension (Figure 2).

*Associations with traffic indicators*. Traffic load in a 100-m buffer was associated with elevated BP in nonmedicated participants with an increase of 0.35 mmHg (95% CI: 0.02, 0.68) systolic and 0.22 mmHg (95% CI: 0.04, 0.40) diastolic BP per 4,000,000 vehicles × m/day, respectively, with no evidence for heterogeneity (Table 4 and Figure 2). The *p*_interaction_ values with BPLM intake were 0.14 and 0.15, respectively for systolic and diastolic BP. No association was found in medicated participants. The estimated odds ratios (ORs) for hypertension and BPLM intake were 1.05 (95% CI: 0.99, 1.11) and 1.04 (95% CI: 0.98, 1.10) per 4,000,000 vehicles × m/day, respectively, with some evidence for heterogeneity for the outcome hypertension (Table 5). In categorical analyses of traffic load and BP, we found the highest effect estimates among the most exposed participants, although no consistent exposure–response relationship was observed (see Supplemental Material, Figure S1). Traffic intensity at the nearest road showed no association with the outcomes (Tables 4, 5 and Figure 2).

*Sensitivity analyses*. Results with right-censored regression (censoring by BPLM use) were similar to those in nonmedicated participants (Table 6). We observed a positive association of traffic load with systolic and diastolic BP. Findings for other pollutants were inconsistent.

**Table 6:** Adjusted^*a*^ associations of TRAP and traffic indicators with systolic and diastolic BP, estimated with right-censored regression and pooled using random-effects meta-analysis.

Outcome and exposure (increment)	Studies (*n*)	Change^*b*^ [mmHg (95% CI)]	*p*_*het*_	*I*^2^
Systolic BP
PM_2.5_ (5 μg/m^3^)	12^*c*^	0.13 (–0.80, 1.07)	0.14	31
PM_2.5_ absorbance (10^–5^/m)	12	0.03 (–0.94, 0.99)	0.06	42
PM_coarse_ (5 μg/m^3^)	12	–0.14 (–0.73, 0.45)	0.03	49
PM_10_ (10 μg/m^3^)	12	–0.06 (–0.57, 0.45)	0.37	8
NO_2_ (10 μg/m^3^)	13	–0.34 (–0.82, 0.13)	0.01	53
NO_x_ (20 μg/m^3^)	13	–0.27 (–0.71, 0.17)	0.00	60
Traffic load (4 × 10^6^ vehicles × m/day)	13^*d*^	0.36 (0.06, 0.67)	0.46	0
Traffic intensity (5,000 vehicles/day)	12^*e*^	0.05 (–0.10, 0.19)	0.72	0
Diastolic BP
PM_2.5_ (5 μg/m^3^)	12^*c*^	0.12 (–0.52, 0.76)	0.05	44
PM_2.5_ absorbance (10^–5^/m)	12	0.24 (–0.23, 0.72)	0.16	29
PM_coarse_ (5 μg/m^3^)	12	0.14 (–0.07, 0.36)	0.35	9
PM_10_ (10 μg/m^3^)	12	0.12 (–0.15, 0.40)	0.63	0
NO_2_ (10 μg/m^3^)	13	0.03 (–0.11, 0.18)	0.58	0
NO_x_ (20 μg/m^3^)	13	0.06 (–0.07, 0.20)	0.55	0
Traffic load (4 × 10^6^ vehicles × m/day)	13^*d*^	0.25 (0.08, 0.42)	0.56	0
Traffic intensity (5,000 vehicles/day)	12^*e*^	0.05 (–0.03, 0.13)	0.60	0
*I*^2^ is a measure of heterogeneity between cohorts, and *p*_het_ is a *p*-value for the *Q*-test of heterogeneity. ^***a***^Adjusted for age, sex, BMI, smoking status, pack-years of smoking, passive smoking, alcohol consumption, physical activity, educational level, economic activity, neighborhood SES (including a random intercept for a neighborhood). ^***b***^Effect estimate refers to the indicated exposure increment. ^***c***^FINRISK, HUBRO, 60-year-olds, SDPP, SNAC-K, TwinGene, EPIC-MORGEN, EPIC-Prospect, HNR, KORA, SAPALDIA (Lugano site), REGICOR; *n *= 91,574. ^***d***^FINRISK, HUBRO, 60-year-olds, SDPP, SNAC-K, TwinGene, EPIC-Umeå, EPIC-MORGEN, EPIC-Prospect, HNR, KORA, SAPALDIA, REGICOR; *n *= 114,648. ^***e***^FINRISK, HUBRO, 60-year-olds, SDPP, SNAC-K, TwinGene, EPIC-Umeå, EPIC-MORGEN, EPIC-Prospect, KORA, SAPALDIA, REGICOR; *n* = 110,033.				

We observed similar effects in the main analysis as compared with the extended analysis, which included DCH and EPIC-Oxford (see Supplemental Material, Figure S2, for PM_2.5_, NO_2_, and traffic load and systolic BP; not shown for other pollutants and diastolic BP; see also forest plots in main and extended meta-analysis with PM_2.5_ and BP in Supplemental Material, Figure S3). When restricting the analysis to cohorts with at least three consecutive BP measurements, we observed a positive association of PM_2.5_ with systolic BP in medicated participants and an increased estimate in nonmedicated participants (see Supplemental Material, Figure S2). No consistent differences by body position during measurement or by the BP recording device were observed.

Increasing the level of adjustment from the crude to the main model increased the effect estimates of PM_2.5_ with systolic BP (see Supplemental Material, Figure S4). Further adjustment with road traffic noise and season in the sensitivity models led to minor decreases in estimates with systolic BP (see Supplemental Material, Figure S5, for PM_2.5_, NO_2_ and traffic load; not shown for other pollutants). Exclusion of participants who had changed their address recently led to a minor decrease in the estimated change in systolic BP with PM_2.5_, increase with NO_x_ and no difference with NO_2_ and traffic load (see Supplemental Material, Figure S5, for PM_2.5_, NO_2_, and traffic load; not shown for NO_x_). Back extrapolation of exposure estimates for PM_10_ and NO_2_ to the time of the baseline examination slightly increased the estimates for PM_10_ and NO_2_ (see Supplemental Material, Figure S5, for NO_2_; not shown for PM_10_). Traffic noise was associated with BP in only some of the cohorts (not shown).

In two-pollutant models including both PM_2.5_ and NO_2_, estimates were higher for PM_2.5_ and more negative for NO_2_ for systolic BP (see Supplemental Material, Table S3). This tendency remained after we excluded six studies with a high correlation of PM_2.5_ and NO_2_ (data not shown). No difference in estimates was observed for diastolic BP (data not shown). A similar but less consistent pattern was observed for PM_10_ and PM_2.5_ absorbance with NO_2_ (data not shown).

In the meta-regression, mean age of the study participants was positively associated with the study-specific estimate for PM_2.5_ and NO_2_ in nonmedicated participants (*p* < 0.05; data not shown); no associations with other study characteristics (including leave-one-out cross-validation *R*^2^ of the LUR model) were found.

## Discussion

In this comprehensive study of up to 15 European population-based cohort studies including up to 164,484 participants, high traffic load in a 100-m buffer around the residence was weakly associated with increased arterial BP in participants who were not taking BPLM, independent of background concentrations of NO_x_ and road traffic noise levels. We also found a positive, yet imprecise, relationship of high traffic load with the odds for hypertension and intake of BPLM. Modeled exposure to PM was not clearly related to BP, although point estimates were mostly elevated. We found positive associations in the subgroup of studies with at least three consequent measurements of BP per participant. Modeled concentrations of NO_x_ were not associated with BP, although we found a weak association between higher NO_2_ and lower BP. Results for PM_2.5_ and NO_2_ were stronger when adjusted for each other.

Living close to a busy road has been positively associated with pulse pressure and inflammation markers (Rioux et al. 2010), impaired cardiac function (Van Hee et al. 2009), narrower retinal arteriolar diameter (Adar et al. 2010), coronary heart disease prevalence and mortality (Gan et al. 2010; Hoffmann et al. 2006), and atherosclerosis progression (Hoffmann et al. 2007; Künzli et al. 2010). We previously reported an increased prevalence of hypertension among participants living near a major road (Fuks et al. 2011). Our results for traffic load in nonmedicated participants were weak, although robust to adjustment for potential confounders such as background air pollution levels, personal cardiovascular risk factors, neighborhood SES, and road traffic noise. We think it is possible that the direct traffic emissions (which are not estimated with LUR, such as ultrafine particles) could be the reason for the observed associations. A relationship between ultrafine particles and acute changes in cardiovascular function—such as heart rate variability, endothelial vasomotor function, and others—was reported in a recent review (Weichenthal 2012). On the other hand, we found no association of traffic intensity on the nearest road with any of the outcomes. This discordance may be explained by the difference between these two variables: whereas traffic intensity pertains to the closest road only (regardless of road type and of other high-traffic roads close by), traffic load takes into account all major roads within 100 m of the residence. As a result, the correlation between the two variables was low to moderate.

We observed positive point estimates of PM with BP in medicated participants and no association in nonmedicated participants. Results for long-term PM_2.5_ in medicated participants were generally in accordance with associations reported in earlier single-cohort studies in adults (Chuang et al. 2011; Coogan et al. 2012; Schwartz et al. 2012), although the confidence intervals were wider in our study despite its large size. The estimates for PM_10_ with BP in medicated participants were similar or even higher (for diastolic BP) compared with those reported in a recent study from China (Dong et al. 2013). Restriction of the analysis to studies with at least three measurements of BP yielded higher estimates for PM in medicated participants. This finding points to the necessity of reducing the outcome measurement error by repeated and standardized assessments of BP. The observed heterogeneity of the results might also be explained in part by different constituents contributing to the complex PM mixture across the European study areas. Recently, Wu et al. (2013) reported positive and inverse short-term associations of different PM constituents with BP.

We found a weak association between higher NO_2_ and lower systolic BP in nonmedicated participants, which, although not statistically significant, was robust to the inclusion of traffic noise and to adjustment for temporal changes by using back-extrapolated concentrations. When we included both PM_2.5_ and NO_2_ in a two-pollutant model for systolic BP, the positive estimate for PM_2.5_ increased in nonmedicated participants, whereas the negative estimate for NO_2_ further decreased. An NO_2_-related BP decrease has been shown before, in a large Danish study using a different exposure model (Sørensen et al. 2012); however, coherent biological explanations are still missing.

We found partially different results in the groups by BPLM intake. Although traffic load was associated with BP in nonmedicated participants, PM was weakly related to BP only in medicated participants. The proportion of medicated participants differed greatly among the studies. A medication-induced decrease in BP may mask any influences of environmental factors, especially if the prescription of BPLM is in part related to environmentally induced high BP. On the other hand, participants not using BPLM may represent a less susceptible population group, especially in older cohorts. It is, therefore, possible that results in nonmedicated participants may underestimate the true effect in the population.

The suggested biological mechanisms for cardiovascular effects of particulate TRAP include the elicitation of local and systemic inflammation and oxidative stress, autonomic imbalance, and endothelial dysfunction (Brook 2007; Brook et al. 2009). Results from animal hypertension models have shown that PM_2.5_ could potentiate hypertension by modulating the sensitivity to pressure stimuli (Sun et al. 2008).

The estimated change in BP after exposure to TRAP is rather small. However, even small changes of arterial BP can be of high public health importance. Reducing the systolic BP by only 2 mm leads to a reduction in stroke mortality of 5%, in coronary heart disease mortality of 4%, and in total mortality of 3% (Whelton et al. 2002). A 2-mmHg reduction in diastolic BP has been linked to a 6% decrease in the risk of coronary heart disease and a 15% decrease in the risk of stroke and transient ischemic attack (Cook et al. 1995).

Assessing exposure with models always implies imprecision (i.e., misclassification), which might have masked or weakened true associations. In addition, TRAP modeling with the ESCAPE protocol was performed on average 5–10 years after BP had been measured. Personal exposure misclassification will likely increase over longer time periods, and possibly mask the small effects. However, in our meta-regression, we did not find any influence of the time period between exposure and outcome assessment on the meta-analysis estimate. In addition, LUR models have been recognized as reliable estimators of spatial air pollution gradients for decades (Eeftens et al. 2011).

Some of the estimated ORs for hypertension and BPLM intake were as high as 1.08 in the present study. However, given the relatively high prevalence rates of BPLM intake of 35–66% across our cohorts, this prevalence OR likely overstates the magnitude of the effect on the prevalence ratio.

One limitation of our study is that BPLM are sometimes prescribed for conditions other than hypertension. For example, beta blockers are also used for managing cardiac arrhythmias. To overcome this limitation, we analyzed several related outcomes, including measured BP only, intake of BPLM only, and hypertension as a composite outcome. Extended outcome definitions, such as prehypertension, could be added to future analyses because prehypertension has been associated with cardiovascular and cerebrovascular disease (Erbel et al. 2012). A more reliable investigation of the air pollution effect in participants using BPLM will be possible in cohorts with repeated prospective assessment of BP and BPLM.

This is by far the largest study to date to investigate the effect of long-term exposure to TRAP on arterial BP and hypertension. We included up to 164,484 participants from large population-based cohorts in Europe. We used the same protocol for dedicated air pollution measurement campaigns and for LUR modeling across all study areas, underwent great efforts to assess and define outcome variables and covariates in comparable ways, and applied identical statistical analysis procedures that accounted for BPLM intake in each cohort. We used data from all ESCAPE cohorts where BP data were available and of satisfactory quality, regardless of whether any effects of air pollution on BP had been investigated or shown in these cohorts previously, therefore diminishing the probability of publication bias.

## Conclusions

This is the largest study on the effect of air pollution on BP and the only meta-analysis to date. Using 15 European population-based cohorts we observed a weak positive association of high residential traffic exposure with arterial BP in participants without BPLM intake and an elevated OR for prevalent hypertension. The relationship of modeled air pollutants with BP was inconsistent, although positive relationships with BP in medicated participants and in the subgroup of studies with higher quality BP measurements were observed. Because of the importance of arterial BP and hypertension as the major risk factors for premature mortality worldwide, these findings have large public health implications and point to the necessity of refined analyses using information on air pollution components, personal characteristics that may convey differential susceptibility, and high-quality outcome assessments.

## Supplemental Material

(6 MB) PDF
