# Supplementary material for: Arterial Blood Pressure and Long-Term Exposure to Traffic-Related Air Pollution: An Analysis in the European Study of Cohorts for Air Pollution Effects (ESCAPE)
Source: Environ Health Perspect. 2014 May 16;122(9):896–905. doi: 10.1289/ehp.1307725 (PMC4154218; doi:10.1289/ehp.1307725)
Supplement: (6 MB) PDF [file ehp.1307725.s001.pdf]

Environmental Health Perspectives doi:10.1289/ehp.1307725

Supplemental Material

Arterial Blood Pressure and Long-Term Exposure to Traffic-Related Air Pollution: An Analysis in the European Study of Cohorts for Air Pollution Effects (ESCAPE)

Kateryna B. Fuks, Gudrun Weinmayr, Maria Foraster, Julia Dratva, Regina Hampel, Danny Houthuijs, Bente Oftedal, Anna Oudin, Sviatlana Panasevich, Johanna Penell, Johan N. Sommar, Mette Sørensen, Pekka Tiittanen, Kathrin Wolf, Wei W. Xun, Inmaculada Aguilera, Xavier Basagaña, Rob Beelen, Michiel L. Bots, Bert Brunekreef, H. Bas Bueno-de-Mesquita, Barbara Caracciolo, Marta Cirach, Ulf de Faire, Audrey de Nazelle, Marloes Eeftens, Roberto Elosua, Raimund Erbel, Bertil Forsberg, Laura Fratiglioni, Jean-Michel Gaspoz, Agneta Hilding, Antti Jula, Michal Korek, Ursula Krämer, Nino Künzli, Timo Lanki, Karin Leander, Patrik K.E. Magnusson, Jaume Marrugat, Mark J. Nieuwenhuijsen, Claes-Göran Östenson, Nancy L. Pedersen, Göran Pershagen, Harish C. Phuleria, Nicole M. Probst-Hensch, Ole Raaschou-Nielsen, Emmanuel Schaffner, Tamara Schikowski, Christian Schindler, Per E. Schwarze, Anne J. Søgaard, Dorothea Sugiri, Wim J.R. Swart, Ming-Yi Tsai, Anu W. Turunen, Paolo Vineis, Annette Peters, and Barbara Hoffmann

Table of Contents **Page**

Methods **3**
1 *Cohort-specific information, funding and acknowledgments* *3*
2 *Exposure assessment* *9*
2.1 Land use regression model 9
2.2 Extrapolation of exposure values back in time 10
2.3 Exposure increments in the analyses 11
2.4 Noise assessment 11
3 *Assessment of blood pressure lowering medication use* *12*
4 *Cohort-specific analysis* *12*
5 *Sensitivity meta-analysis and meta-regression* *13*

Supplemental Tables **16**
Table S1. Availability of exposure and outcome variables in the analysis cohorts **16**
Table S2. Cohort-specific correlation coefficients of long-term traffic-related exposure values (Spearman's rho) **17**

Table S3. Adjusted association of traffic-related air pollution (5-µg/m³ increase in $PM_{2.5}$, or 10-µg/m³ increase in NO_2) with systolic blood pressure (with or without BPLM intake, change in mmHg), prevalent hypertension (odds ratio), and use of blood pressure lowering medication (odds ratio) in the study population, based on multi-pollutant models (N=91,574) **18**

Supplemental Figures **19**

Figure S1. Meta-analysis with traffic load at the major road fragments in categories with systolic and diastolic blood pressure **19**

Figure S2. Association of $PM_{2.5}$, NO_2, and traffic load at the major road fragments with systolic blood pressure in main and extended meta-analysis, and stratified by details of measurement procedure **20**

Figure S3. Cohort-specific and meta-analysis estimates of association of $PM_{2.5}$ with systolic blood pressure: main and extended meta-analysis, stratified by medication status **22**

Figure S4. Association of $PM_{2.5}$ with systolic blood pressure: a comparison of adjustment models from crude to main in medicated and non-medicated with blood-pressure lowering medication, and censored regression **23**

Figure S5. Extended adjustment sets and analysis with back-extrapolated exposures with systolic blood pressure in participants not medicated with blood pressure lowering medication **24**

References **26**

Methods

1 Cohort-specific information, funding and acknowledgments

The National FINRISK Study (FINRISK, Finland) has been established in 1972; randomly selected representative population samples are recruited from different parts of Finland every five years. For this study, three cross-sectional population surveys (1997, 2002, and 2007) and two study areas (cities of Helsinki/Vantaa and Turku/Loimaa region) were used (Peltonen et al. 2008). *Funding and acknowledgments.* For the Finnish part, additional funding came from the Academy of Finland (project number 129317). Exposure assessment was performed by Tarja Yli-Tuomi, Pekka Taimisto, and Arto Pennanen from the Department of Environmental Health. Mortality, area-level SES, and building data were provided by Statistics Finland.

The Danish Cancer study (DCH, Denmark) aims to investigate diet and lifestyle in relation to the incidence of cancer and other chronic diseases. n total, 57,053 of 160,725 residents of Copenhagen or Aarhus aged 50 – 64 years without a history of cancer (excluding non-melanoma skin cancer) were enrolled into the original cohort between 1993 and 1997 (Tjønneland et al. 2007). Only participants from the Copenhagen area were included in ESCAPE. *Funding and acknowledgments.* The Danish Cancer Society contributed funding to establish the Diet Cancer and Health study.

The population-based Oslo Health Study (HUBRO, Norway) was designed to identify health needs within Oslo and determine the priorities of the health sector (Søgaard et al. 2004). Another aim was to identify social and geographical differences in health and associated risk factors. HUBRO was carried out in the city of Oslo in 2001. All men and women born in 1924, 1925, 1940, 1941, 1955, 1960 and 1970, who resided in Oslo on December 31, 1999, were invited. 18 770 individuals participated in the study. *Funding and acknowledgments.* The data collection

was conducted as part of the Oslo Health Study 2000-2001 in collaboration with the Norwegian Institute of Public Health. The authors would like to thank Jon Wickmann for his efforts to coordinate the Oslo group and to assure the quality of exposure assessment in the HUBRO cohort.

The Stockholm 60-year olds cohort (60-year-olds, Sweden) was initiated for identification of biological and socio-economic risk factors and predictors for cardiovascular diseases through a cross-sectional health screening study (Wändell et al. 2007). A total of 4106 (2779 men and 2681 women) randomly selected participants born between 1 July 1937 and 30 June 1938, and living in Stockholm County, participated. *Funding and acknowledgments.* The financial support for the combined work with the Stockholm studies was provided by the Swedish Environmental Protection Agency, the Swedish Heart-Lung Foundation and the Swedish Council for Working Life and Social Research. The study was funded by the Swedish Research Council (09533), the Stockholm County Council, and the Swedish Heart-Lung Foundation.

Stockholm diabetes preventive program (SDPP, Sweden) aims to initiate and develop methods for type 2-diabetes prevention and also study the etiology of type 2 diabetes (Eriksson et al. 2008). SDPP is a population-based cross-sectional study which at base line comprised of 3,128 men and 4,821 women aged 35-56 years, half with a family history of diabetes. The base line study for men was done between 1992–1994 and for women 1996–1998. The participants were chosen from within 5 municipalities in Stockholm County. *Funding and acknowledgments.* The financial support for the combined work with the Stockholm studies was provided by the Swedish Environmental Protection Agency, the Swedish Heart-Lung Foundation and the Swedish Council for Working Life and Social Research. The study was supported by grants from

Stockholm County Council, Swedish Council for Working Life and Social Research, Swedish Research Council, Swedish Diabetes Foundation, Novo Nordisk Scandinavia.

The Swedish National study of Aging and Care in Kungsholmen (SNAC-K, Sweden) is an ongoing longitudinal study with the ultimate goal of contributing to the understanding of the ageing process and identifying possible preventive strategies to improve health and care in elderly adults (Lagergren et al. 2004). The study population consists of 3363 randomly sampled 60 year and older individuals in a central area of Stockholm (Kungsholmen) during a 3-year period (March 2001- June 2004). *Funding and acknowledgments.* The financial support for the combined work with the Stockholm studies was provided by the Swedish Environmental Protection Agency, the Swedish Heart-Lung Foundation and the Swedish Council for Working Life and Social Research.

The Swedish Twin Registry (TwinGene, Sweden) is a study of twins residing in Stockholm County from Swedish National Twin Registry (Magnusson et al. 2013). In TwinGene, twins born before 1958 were contacted in the years 2004-2008 for the collection of biological samples. The study focused on the importance of quantitative trait loci and environmental factors for cardiovascular disease. *Funding and acknowledgments.* The financial support for the combined work with the Stockholm studies was provided by the Swedish Environmental Protection Agency, the Swedish Heart-Lung Foundation and the Swedish Council for Working Life and Social Research.

We included four sub-cohorts from the European Prospective Investigation into Cancer and Nutrition (EPIC) (Riboli et al. 2002). EPIC is a large multi-center prospective cohort study,

involving 23 European centers in 10 countries in Western Europe. EPIC was designed to study the relationship between diet and the risk of chronic diseases, particularly cancer.

EPIC-Umeå is a sub-cohort of EPIC in Sweden, It joined the EPIC study in 1996–1997 and consists primarily the Västerbotten Intervention Cohort (Oudin et al. 2012). Since 1985 all individuals 40, 50 and 60 years of age (with subsets at age of 30 and 70) in the population of the county were invited for screening within the VIP cohort. The baseline screening was performed in 1992–1996. *Funding and acknowledgments.* We wish to acknowledge The Swedish Cancer Society and the Swedish Scientific Council for funding, and the Västerbotten County Council for giving access to data.

Two EPIC sub-cohorts in the Netherlands were included:

- The EPIC Monitoring Project on Risk Factors for Chronic Diseases (EPIC-MORGEN, the Netherlands) cohort consists of a general population sample of 10 260 men and 12 394 women aged 20–59 years from three Dutch towns (Amsterdam, Doetinchem and Maastricht). From 1993 to 1997 each year a new random sample, consisting of 6000 participants, was examined (Beulens et al. 2010).
- The EPIC-Prospect study (the Netherlands) consists of 17,500 healthy women, living in Utrecht and surroundings were enrolled. Women were recruited from breast cancer screening participants, age 50-70 years at enrolment. Baseline information was collected between 1993-1997 on the basis of two self-administered questionnaires and a medical examination (Beulens et al. 2010).
- *Funding and acknowledgments.* The EPIC-MORGEN and EPIC-PROSPECT cohorts were supported by the Dutch Ministry of Public Health, Welfare and Sports (VWS),

Dutch Prevention Funds, Dutch ZON (Zorg Onderzoek Nederland), and Statistics Netherlands.

The EPIC Oxford cohort (EPIC-Oxford, the United Kingdom) was recruited from the nationwide general population throughout the United Kingdom into the Oxford cohort, containing 65,429 men and women through medical general practices or by post between 1993 and 2000 (Danesh et al., 2007).

The Heinz Nixdorf Risk Factors, Evaluation of Coronary Calcification, and Lifestyle (Recall) study (HNR, Germany) is an ongoing population-based, prospective cardiovascular cohort study that started in 2000. The main objective of the study is the assessment of the predictive power of coronary artery calcification for the incidence of hard coronary events such as myocardial infarction and sudden cardiac death in an unselected general population. HNR includes 4814 randomly selected participants aged 45 to 75 years from three large adjacent German cities (Essen, Mülheim, Bochum) of the densely populated and highly industrialized Ruhr Area (Schmermund et al. 2002; Stang et al. 2006). *Funding and acknowledgments.* We thank all study participants and the dedicated personnel of the Heinz Nixdorf Recall Study. We gratefully acknowledge the collaboration with K.-H. Jöckel, D. Grönemeyer, R. Seibel, K. Mann, L. Vollbracht, K. Lauterbach. We thank the North Rhine-Westphalia State Agency for Nature, Environment and Consumer Protection for providing road maps with traffic data and emission data from the reference sites for back-extrapolation. The study was supported by the Heinz Nixdorf Foundation [chairman: M. Nixdorf; former chairman: G. Schmidt (deceased)], the German Ministry of Education and Science, the German Research Foundation (DFG; projects JO-170/8-1, HO 3314/2-1, SI 236/8-1, and SI236/9-1).

The Cooperative Health Research in the Region of Augsburg (KORA, Germany) study is conducted within the framework of the Cooperative Health Research in the Region of Augsburg and consists of four cross-sectional, population-representative surveys (Holle et al. 2005). Main objectives of KORA to assess health indicators (morbidity, mortality) and health care (utilization, costs), quantify prevalence of risk factors for cardiovascular and other chronic diseases, and study the impact of lifestyle, metabolic and genetic factors in the etiology of cardiovascular and other chronic diseases. ESCAPE includes two of the surveys, conducted in 1994-1995 and 1999-2001 in the city of Augsburg and two adjacent rural counties. *Funding and acknowledgments.* The KORA research platform and the MONICA Augsburg studies were initiated and financed by the Helmholtz Zentrum München, German Research Center for Environmental Health, which is funded by the German Federal Ministry of Education and Research and the State of Bavaria.

The Swiss Study on Air Pollution and Lung and Heart Disease In Adults (SAPALDIA, Switzerland) consists of a random population sample across 8 geographic areas (Aarau, Basel, Davos, Geneva, Lugano, Montana, Payerne and Wald) that was obtained in 1991 (Ackermann-Liebrich et al. 2005). The main aim of the study is to assess the effect of air pollution (outdoor and indoor) on respiratory and cardiovascular health, with a special focus on how the respiratory and cardio-vascular systems interact in this regard, and on the role of lifestyle and genetic background. For the current analyses, data from SAPALDIA follow-up in 2002 from Basel, Lugano and Geneva were used. *Funding and acknowledgments.* The study was supported by the Swiss National Science Foundation (grants no 33CSCO-134276/1, 33CSCO-108796, 3247BO-104283, 3247BO-104288, 3247BO-104284, 3247-065896, 3100-059302, 3200-052720, 3200-042532, 4026-028099), the Federal Office for Forest, Environment and Landscape, the Federal

Office of Public Health, the Federal Office of Roads and Transport, the canton's government of Aargau, Basel-Stadt, Basel-Land, Geneva, Luzern, Ticino, Valais, and Zurich, the Swiss Lung League, the canton's Lung League of Basel-Stadt/Basel Landschaft, Geneva, Ticino, Valais and Zurich, SUVA, Freiwillige Akademische Gesellschaft, UBS Wealth Foundation, Talecris Biotherapeutics GmbH, Abbott Diagnostics, European Commission 018996 (GABRIEL), Wellcome Trust WT 084703MA. The study could not have been done without the help of the study participants, technical and administrative support and the medical teams and field workers at the local study sites.

REGICOR (Registre Gironí del Cor – Girona's heart registry) is a large study investigating risk factors of cardiovascular diseases in the Spanish region of Girona (Grau et al. 2007). REGICOR consists of a cohort of 4,804 participants recruited in 1995 (Cohort1995) and 2000 (Cohort2000), aged 25 to 74 at baseline. The cohort is extracted from a specific geographic region in the North-East of Spain, but is representative for the cardiovascular health of Spanish populations at large. Cohort2000 was included in the current analysis. *Funding and acknowledgments.* This study was funded by grants from the Fondo de Investigación Sanitaria-Instituto Carlos III/European Development Research Funds (Red de Investigación Cardiovascular RD12/0042/0013, RD12/0042/0061, FIS PI081327, FIS C03/09, FIS C03/045), Fundació Marato TV3 (81630), and the Catalan Government (SGR 2009 SGR1195).

2 Exposure assessment

2.1 Land use regression model

Concentrations of particulate matter (PM), including particles with diameter ≤2.5 μm ($PM_{2.5}$), ≤10 μm (PM_{10}), >2.5 to ≤10 μm (PM_{coarse}; calculated as PM_{10} minus $PM_{2.5}$), $PM_{2.5}$ absorbance, and nitrogen oxides (NO_2 – nitrogen dioxide and NO_x – nitrogen oxide) at residence were

estimated in each study region using Land Use Regression (LUR) models, based on a harmonized approach described elsewhere (Beelen et al. 2013; Eeftens et al. 2012a). Measurements of both PM and nitrogen oxides were conducted for a one-year period between 2008 and 2011 at sites chosen to represent the anticipated spatial variation of air pollution in the corresponding study area (Cyrys et al. 2012; Eeftens et al. 2012b). Measurements of NO_x were conducted in all participating regions. PM was measured in the Lugano site of the SAPALDIA study and not measured in the other two participating SAPALDIA sites (Basel and Geneva). PM was not measured in the European Prospective Investigation into Cancer and Nutrition (EPIC) in Umeå (EPIC-Umeå, Sweden). Annual averages of measured pollutant concentrations at the monitoring sites and predictor variables, derived from Europe-wide and local Geographic Information System databases were used to develop LUR models in each ESCAPE study area (Beelen et al. 2013; Eeftens et al. 2012a). Concentrations for each individual's residence were then predicted using the developed study-specific LUR models, and the extreme values were truncated to the minimum and maximum measured values at the monitoring sites.

2.2 Extrapolation of exposure values back in time

The predicted concentrations for PM_{10} and NO_2 were back extrapolated to the time of the BP measurement, if corresponding measured data from routine network sites were available. The following standard procedure was employed: we divided the mean concentration of the respective pollutant (PM_{10} or NO_2) at the baseline period (2-year mean: 12 months before and 12 months after the baseline measurement of the participant), measured by the routine monitoring station, by the respective annual concentration of PM_{10} (or NO_2) of the year, when measurements for the land use regression (LUR) model were performed. With this ratio, we multiplied the corresponding modeled LUR concentration to obtain the back extrapolated value. It was possible

to perform back extrapolation of PM_{10} values in 7 cohorts (TwinGene, EPIC-PROSPECT, EPIC-MORGEN, EPIC-Oxford, HNR, KORA, REGICOR), and of NO_2 values in 13 cohorts (DCH, 60-year-olds, SDPP, SNAC-K, TwinGene, EPIC-Umeå, EPIC-Oxford, EPIC-PROSPECT, EPIC-MORGEN, HNR, KORA, SAPALDIA, REGICOR). In cohorts with recent measurements (FINRISK, HUBRO), temporal adjustment of exposure values was not performed. Further details on the back-extrapolation method can be found at the study website (ESCAPE 2012).

2.3 Exposure increments in the analyses

All exposure increments were a priori chosen centrally for the complete ESCAPE study to ensure that all papers in ESCAPE would report results on the same scale. The standardized increments were based on the mean 5th-95th percentile difference across all ESCAPE study areas. Results were calculated for fixed increments of exposures: 5 µg/m³ for $PM_{2.5}$ and PM_{coarse}, 10 µg/m³ for PM_{10} and NO_2, 20 µg/m³ for NO_x, and 10^{-5} m^{-1} for $PM_{2.5}$ absorbance. Traffic load on major road fragments in a 100 m buffer was included in the analyses as a continuous variable with an increment of 4,000,000 vehicles × m/day, as well as in categories: ≤500,000 (reference); >500,000 & ≤1,500,000; >1,500,000 & ≤3,000,000; >3,000,000 vehicles × m/day. Traffic intensity at the nearest road was analyzed as a continuous variable with an increment of 5,000 vehicles/day.

2.4 Noise assessment

Since 2002 member states of the European Union (EU) are obliged to produce every 5th year noise maps for major roads, major railways and major airports and for larger agglomerations. ESCAPE made use of data from local assessments carried out for the first round of noise mapping in the EU (European Commission 2002). For cohorts where the EU noise maps were not available, (additional) noise calculations were carried out in accordance with the EU-

Directive 2002/49/EC (European Commission 2002). The noise level (L_{den}: day-evening-night equivalent level) was calculated for the most exposed facade of dwellings. National calculations methods were used in the study areas of the Finnish, Swedish, Norwegian, Danish, Swiss, Dutch and German cohorts; the interim method of the EU was applied for REGICOR.

3 Assessment of blood pressure lowering medication use

Intake of blood pressure (BP) lowering medication (BPLM) at the time of a baseline examination was assessed by questionnaire or interview in all studies but EPIC-Oxford. Owing to this, EPIC-Oxford was not included in the main analysis.

To categorize BPLM, five cohorts (HUBRO, 60-year-olds, HNR, KORA, and SAPALDIA) used the Anatomical Therapeutic Chemical Classification System of the Collaborating Centre for Drug Statistics Methodology (WHO 2006): diuretics, β-blockers, angiotensin-converting enzyme inhibitors, angiotensin-receptor antagonists, calcium-channel blockers, α-blockers, centrally active antihypertensive drugs, and hydralazine. The remaining cohorts used pre-defined study-specific categories.

4 Cohort-specific analysis

The main model fit and assumptions for linear regression were checked using a centrally developed code: collinearity of predictor variables; distribution of residuals; presence of influential observations. These checks were performed in the model without exposure.

The following model specifications were used in the cohort-specific analyses:

- Model 1: crude (only exposure); fixed-effects model.
- Model 2: adjusted for sex, age (centered), age (centered) squared; fixed-effects model.

- Model 3: adjusted for sex, age (centered), age(centered) squared, BMI (centered), BMI (centered) squared, smoking status, pack-years of smoking (centered), pack-years of smoking (centered) squared, any passive smoking, weekly total alcohol consumption; wine consumption as drinks per week (if available; centered), physical activity, educational level, economic activity; fixed-effects model.
- Model 3a: same covariates as in model 3; random-effects model (neighborhood as random intercept)
- Model 4 (main model): model 3a with neighborhood level SES; random-effects model.

If the random-effects models did not converge, we accounted for clustering by using the robust variance estimator for the cluster (neighborhood), specifying that the standard errors allow for intragroup correlation, relaxing the usual requirement that the observations be independent. In the TwinGene cohort, consisting of twin pairs by about 50%, a random intercept was introduced for twin pair instead (based on the lower AIC).

5 Sensitivity meta-analysis and meta-regression

To study the effect of the quality of BP measurement on the estimate, we combined studies in groups according to details of the BP measurement procedure and performed the stratified meta-analysis. First, we divided the studies in 3 groups by the number of consequent measurements:

- Group 1: ≥3 BP measurements (HUBRO, SNAC-K, HNR, KORA included);
- Group 2: ≥2 BP measurements (FINRISK, 60-year-olds, EPIC-Umeå, EPIC-MORGEN, EPIC-Prospect, SAPALDIA, REGICOR added to the studies in Group 1);
- Group 3: <2 measurements (SDPP; DCH, EPIC-Oxford added to the studies in Group 2).

Next, we divided studies by participant's position during the BP measurement:

- Group 1: sitting position (FINRISK, SDPP, TwinGene, EPIC-Oxford, HNR, KORA, REGICOR);
- Group 2: supine position (60-year-olds, EPIC-MORGEN, EPIC-Prospect, and DCH).

Studies where BP was not measured in the same position in all participants (EPIC-Umeå, SNAC-K) were excluded.

Finally, we divided studies by the device used for BP measurement:

- Group 1: using an automated oscillometric device (AOD; included HUBRO, 60-year-olds, EPIC-MORGEN, EPIC-Prospect, HNR, SAPALDIA, REGICOR, DCH, EPIC-Oxford);
- Group 2: using a sphygmomanometer (SM; included FINRISK, SDPP, SNAC-K, EPIC-Umea). Two cohorts where both AOD and SM were used (TwinGene, KORA) were excluded. HNR, where for the few participants the missing values with AOD were replaced by measurements with SM, was included in Group 1.

We carried meta-regression with the metareg command in STATA 12.0, using as the following parameters independent predictors:

- time interval between BP measurement and exposure assessment (year of exposure measurement minus a) first and b) last year of BP measurement, entered as continuous variable, or in 5 year categories, and in 10-year categories;
- mean and standard deviation of LUR exposure;
- geographical position: North, Center, South;
- proportion of participants with road traffic noise levels at the residence > 65 dB,

- cohort characteristics: mean and standard deviation of BP, age, BMI, proportion of participants under BP lowering treatment, with prevalent hypertension, current smokers, frequent alcohol drinkers, practicing no sport, unemployed, with low education.

Meta-regression was performed for systolic BP in medicated and non-medicated participants with $PM_{2.5}$ and NO_2 as exposures. The significance level was set to $p < 0.01$ to account for the multiple testing.

Supplemental Tables

Table S1. Availability of exposure and outcome variables in the analysis cohorts.

Cohort	PM exposures[a]	Nitrogen oxides[b]	Traffic load[c]	Traffic intensity[d]	Traffic noise	BP	BPLM	Hypertension	Included in the main analysis	Included in the extended analysis
FINRISK	yes	yes	yes	yes	yes	yes	yes	yes	yes	yes
DCH	yes	yes	yes	yes	yes	yes	yes	yes	no[e]	yes
HUBRO	yes	yes	yes	yes	yes	yes	yes	yes	yes	yes
60-year-olds	yes	yes	yes	yes	yes	yes	yes	yes	yes	yes
SDPP	yes	yes	yes	yes	no	yes	yes	yes	yes	yes
SNAC-K	yes	yes	yes	yes	yes	yes	yes	yes	yes	yes
TwinGene	yes	yes	yes	yes	yes	yes	yes	yes	yes	yes
EPIC-Umeå	no[f]	yes	yes	yes	no	yes	yes	yes	yes	yes
EPIC-MORGEN	yes	yes	yes	yes	yes	yes	yes	yes	yes	yes
EPIC-Prospect	yes	yes	yes	yes	yes	yes	yes	yes	yes	yes
EPIC-Oxford	yes	yes	yes	yes	no	yes	no	yes	no	yes
HNR	yes	yes	yes	no	yes	yes	yes	yes	yes	yes
KORA	yes	yes	yes	yes	yes	yes	yes	yes	yes	yes
SAPALDIA	yes[g]	yes	yes	yes	yes	yes	yes	yes	yes	yes
REGICOR	yes	yes	yes	yes	yes	yes	yes	yes	yes	yes

BP = blood pressure; BPLM = blood pressure lowering medication.

[a]$PM_{2.5}$, $PM_{2.5}$ absorbance, PM_{coarse}, PM_{10}. [b]NO_2, NO_x. [c]Traffic load in the major road fragments in a 100 m buffer around participant's address. [d]Traffic intensity on the nearest road. [e]Excluded due to different recording of BP values for hypertensive and non-hypertensive participants. [f]PM measurements were not performed in EPIC-Umeå. [g]PM exposures are available only for the Lugano site of SAPALDIA.

Table S2. Cohort-specific correlation coefficients of long-term traffic-related exposure values (Spearman's rho).

Exposure	FINRISK	DCH	HUBRO	60-year-olds	SDPP	SNAC-K	Twin Gene	EPIC-Umeå	EPIC-MORGEN	EPIC-Prospect	EPIC-Oxford	HNR	KORA	SAPALDIA	REGICOR
$PM_{2.5}$ × $PM_{2.5abs.}$	0.99	0.48	0.47	0.8	0.63	0.8	0.8	–	0.63	0.64	0.61		0.43	0.65	0.82
$PM_{2.5}$ × PM_{coarse}	0.02	0.62	0.14	0.77	0.59	0.77	0.77	–	0.24	0.73	0.15	0.68	0.28	0.52	0.39
$PM_{2.5}$ × PM_{10}	0.67	0.72	0.71	0.7	0.52	0.7	0.66	–	0.32	0.61	0.55	0.88	0.38	0.64	0.71
$PM_{2.5}$ × NO_2	0.41	0.64	0.4	0.75	0.59	0.75	0.75	–	0.19	0.37	0.88	0.64	0.36	0.67	0.67
$PM_{2.5}$ × traffic load	0.27	0.35	0.21	0.49	0.2	0.49	0.47	–	0.22	0.32	0.32	0.21	0.25	0.38	0.35
$PM_{2.5}$ × traffic noise	0.24	0.29	0.3	0.62	–	0.62	0.58	–	0.13	0.38	–	0.29	0.35	0.31	0.65
$PM_{2.5abs.}$ × PM_{coarse}	0.08	0.59	0.21	0.96	0.95	0.96	0.95	–	0.73	0.68	0.37	0.73	0.83	0.73	0.32
$PM_{2.5abs.}$ × PM_{10}	0.69	0.69	0.21	0.92	0.84	0.92	0.89	–	0.88	0.97	0.54	0.9	0.67	0.59	0.79
$PM_{2.5abs.}$ × NO_2	0.45	0.71	0.78	0.91	0.87	0.91	0.91	–	0.77	0.84	0.76	0.63	0.67	0.69	0.88
$PM_{2.5abs.}$ × traffic load	0.33	0.5	0.34	0.57	0.24	0.57	0.57	–	0.51	0.43	0.38	0.39	0.43	0.21	0.39
$PM_{2.5abs.}$ × traffic noise	0.29	0.45	0.34	0.73	–	0.73	0.71	–	0.36	0.49	–	0.47	0.45	0.18	0.76
PM_{10} × PM_{coarse}	0.71	0.64	0.38	0.95	0.85	0.95	0.92	–	0.89	0.68	0.78	0.69	0.77	0.76	0.51
PM_{10} × NO_2	0.65	0.81	0.28	0.91	0.88	0.91	0.88	–	0.9	0.83	0.49	0.54	0.69	0.81	0.78
PM_{10} × traffic load	0.41	0.51	0.38	0.57	0.25	0.57	0.55	–	0.53	0.39	0.29	0.2	0.26	0.35	0.52
PM_{10} × traffic noise	0.17	0.43	0.29	0.7	–	0.7	0.64	–	0.44	0.46	–	0.31	0.31	0.35	0.73
NO_x × NO_2	0.88	0.99	0.94	0.92	0.76	0.91	0.91	0.51	0.92	0.87	0.93	0.93	0.76	0.92	0.99
NO_2 × PM_{coarse}	0.55	0.72	0.43	0.91	0.85	0.91	0.9	–	0.89	0.63	0.14	0.46	0.8	0.8	0.29
NO_2 × traffic load	0.42	0.65	0.47	0.54	0.22	0.54	0.53	0.46	0.52	0.37	0.36	0.56	0.37	0.16	0.35
NO_2 × traffic noise	0.3	0.62	0.47	0.7	–	0.7	0.65	–	0.43	0.38	–	0.36	0.44	0.23	0.62

Table S3. Adjusted association of traffic-related air pollution (5-µg/m³ increase in $PM_{2.5}$, or 10-µg/m³ increase in NO_2) with systolic blood pressure (with or without BPLM intake, change in mmHg), prevalent hypertension (odds ratio), and use of blood pressure lowering medication (odds ratio) in the study population, based on multi-pollutant models (N=91,574).

**Outcome and exposure**	**Adjusted for**	**Estimate (95% CI)**	**$p_{(het.)}$**	**I^2**
**Systolic BP, no BPLM**				
$PM_{2.5}$	NO_2	0.76 (-0.34, 1.86)	0.06	42
$PM_{2.5}$	NO_2, traffic noise	0.49 (-0.59, 1.57)	0.24	21
NO_2	$PM_{2.5}$	-0.58 (-1.19, 0.03)	0.00	62
NO_2	$PM_{2.5}$, traffic noise	-0.54 (-1.11, 0.03)	0.01	56
**Systolic BP, BPLM intake**				
$PM_{2.5}$	NO_2	1.3 (-0.76, 3.36)	0.19	26
$PM_{2.5}$	NO_2, traffic noise	1.63 (-1.68, 4.94)	0.02	53
NO_2	$PM_{2.5}$	-0.53 (-1.57, 0.51)	0.08	39
NO_2	$PM_{2.5}$, traffic noise	-0.57 (-1.24, 0.1)	0.38	7
**Hypertension**				
$PM_{2.5}$	NO_2	1.11 (1.00, 1.24)	0.29	16
$PM_{2.5}$	NO_2, traffic noise	1.08 (0.90, 1.29)	0.08	41
NO_2	$PM_{2.5}$	0.98 (0.92,1.04)	0.07	41
NO_2	$PM_{2.5}$, traffic noise	0.98 (0.94,1.02)	0.62	0
**BPLM use**				
$PM_{2.5}$	NO_2	1.09 (0.98, 1.23)	0.83	0
$PM_{2.5}$	NO_2, traffic noise	1.12 (0.98, 1.27)	0.42	2
NO_2	$PM_{2.5}$	1.01 (0.95, 1.06)	0.22	23
NO_2	$PM_{2.5}$, traffic noise	1.00 (0.92, 1.09)	0.05	45

BP = blood pressure; BPLM = BP lowering medication; 95% CI = confidence interval at $\alpha = 0.05$; I^2 = measure of heterogeneity between cohorts; $p_{(het.)}$ = p-value for the Q-test of heterogeneity.

Adjusted for age, sex, body mass index, smoking status, pack-years, passive smoking, alcohol consumption, physical activity, educational level, economic activity, neighborhood SES (including a random intercept for neighborhood). Cohorts included in the multipollutant analyses: FINRISK, HUBRO, 60-year-olds, SDPP, SNAC-K, TwinGene, EPIC-MORGEN, EPIC-Prospect, HNR, KORA, SAPALDIA (Lugano site), REGICOR. Excluded cohorts: DCH, EPIC-Oxford, EPIC-Umeå; SDPP in models with noise).

Supplemental Figures

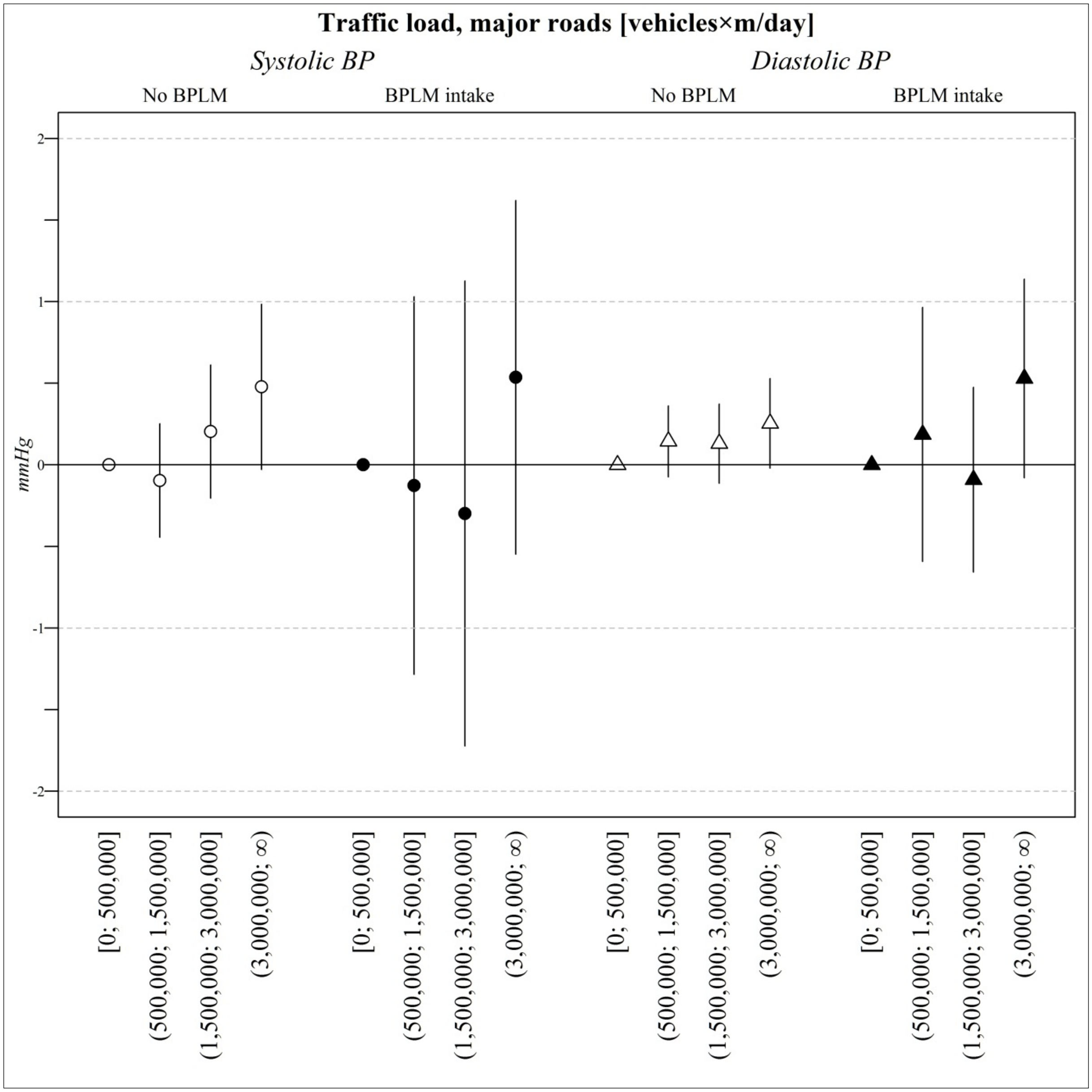

Figure S1. Meta-analysis with traffic load at the major road fragments in categories with systolic and diastolic blood pressure. Legend: BP = blood pressure; BPLM = BP lowering medication; change in mmHg with 95% confidence interval is presented. Adjusted for age, sex, body mass index, smoking status, pack-years, passive smoking, alcohol consumption, physical activity, educational level, economic activity, neighborhood SES (including a random intercept for neighborhood). Cohorts included: FINRISK, HUBRO, 60-year-olds, SDPP, SNAC-K, TwinGene, EPIC-Umeå, EPIC-MORGEN, EPIC-Prospect, HNR, KORA, SAPALDIA, REGICOR.

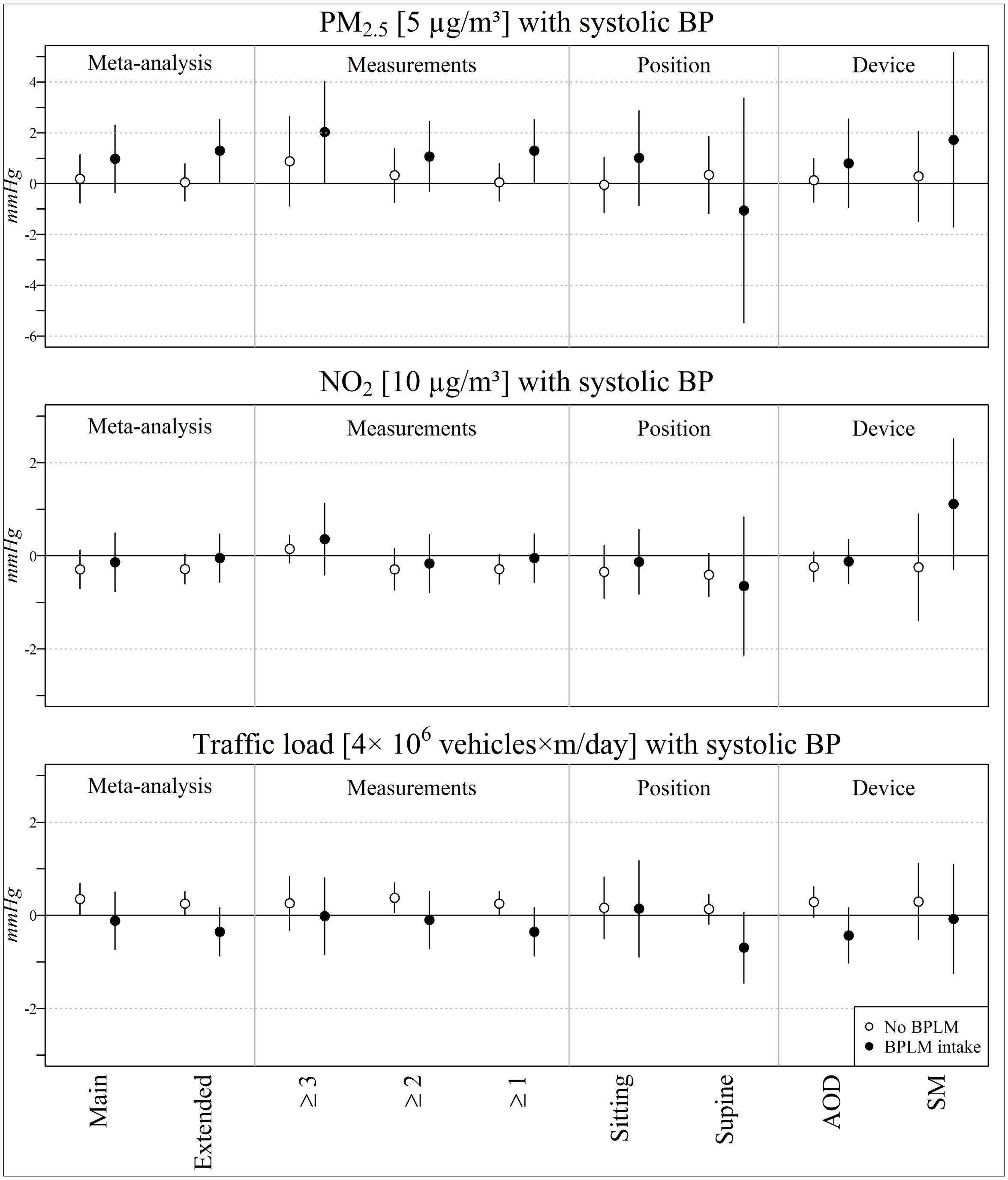

Figure S2. Association of $PM_{2.5}$, NO_2, and traffic load at the major road fragments with systolic blood pressure in main and extended meta-analysis, and stratified by details of measurement procedure. Legend: BP = blood pressure; BPLM = BP lowering medication; AOD = automated oscillometric device; SM = sphygmomanometer; change in mmHg with 95% confidence interval is presented.

Cohorts in the main meta-analysis (N=13): FINRISK, HUBRO, 60-year-olds, SDPP, SNAC-K, TwinGene, EPIC-Umeå[a], EPIC-MORGEN, EPIC-Prospect, HNR, KORA, SAPALDIA (only Lugano site with $PM_{2.5}$), REGICOR.

In the extended meta-analysis, DCH and EPIC-Oxford were added (N=15).

Stratification by number of consecutive BP measurements:

- ≥3 (N=4): HUBRO, SNAC-K, HNR, KORA included,
- ≥2 (N=12): FINRISK, 60-year-olds, TwinGene, EPIC-Umeå[a], EPIC-MORGEN, EPIC-Prospect, SAPALDIA, REGICOR added
- ≥1 (N=15): SDPP, DCH, EPIC-Oxford added.

Stratification by position during measurement:

- Sitting (N=7): FINRISK, SDPP, TwinGene, EPIC-Oxford, HNR, KORA, REGICOR included;
- Supine (N=4): 60-year-olds, EPIC-MORGEN, EPIC-Prospect, DCH included;

Stratification by device:

- AOD (N=8): HUBRO, 60-year-olds, DCH, EPIC-MORGEN, EPIC-Prospect, HNR, SAPALDIA, REGICOR included;
- SM (N=4): FINRISK, SDPP, SNAC-K, EPIC-Umeå[a] included.

[a]EPIC-Umeå was included only in the analyses with NO_2 and traffic load at major road fragments, because $PM_{2.5}$ was not modeled in this cohort.

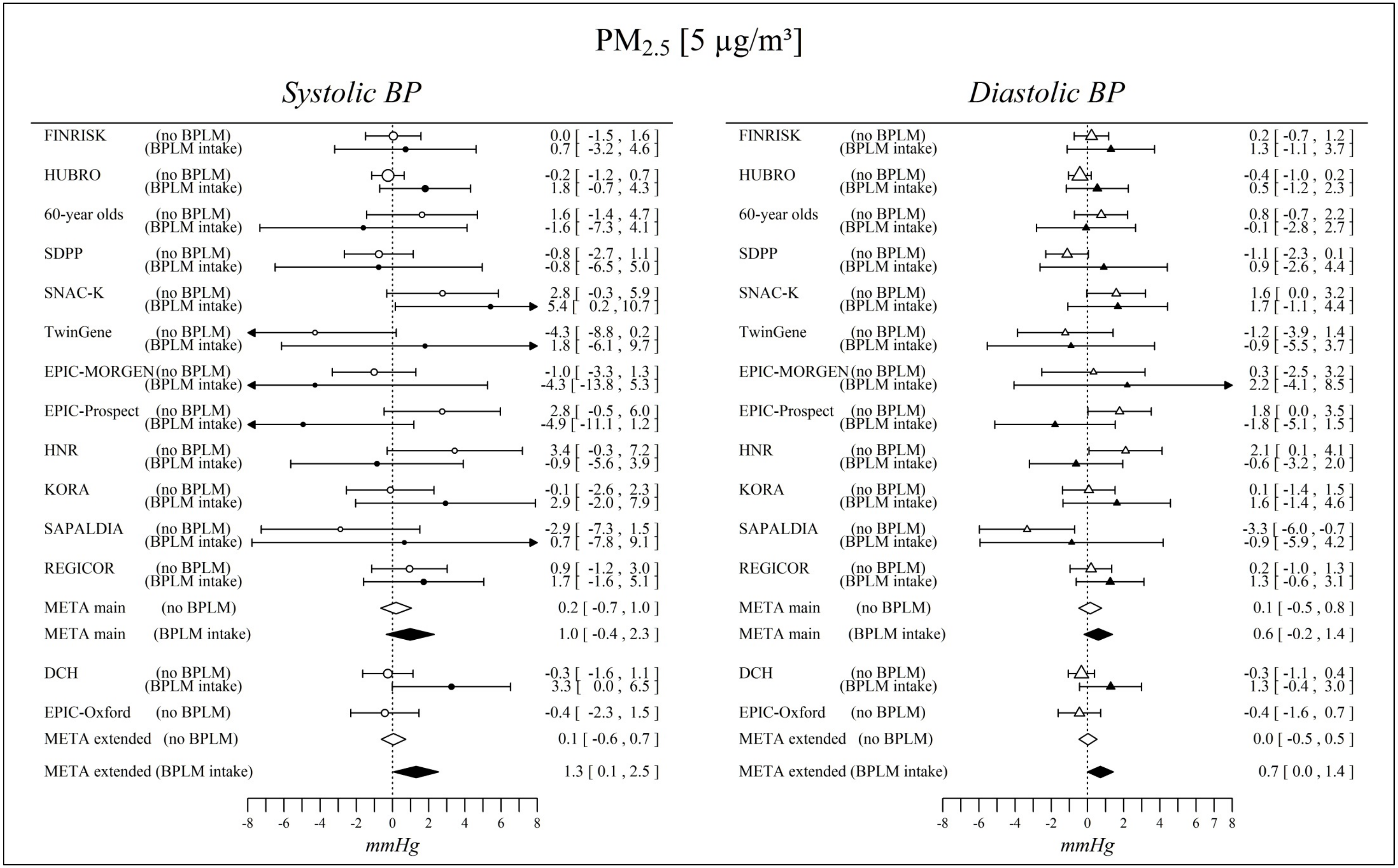

Figure S3. Cohort-specific and meta-analysis estimates of association of $PM_{2.5}$ with systolic blood pressure: main and extended meta-analysis, stratified by medication status. Legend: BP = blood pressure; BPLM = BP lowering medication; change in mmHg with 95% confidence interval is presented.

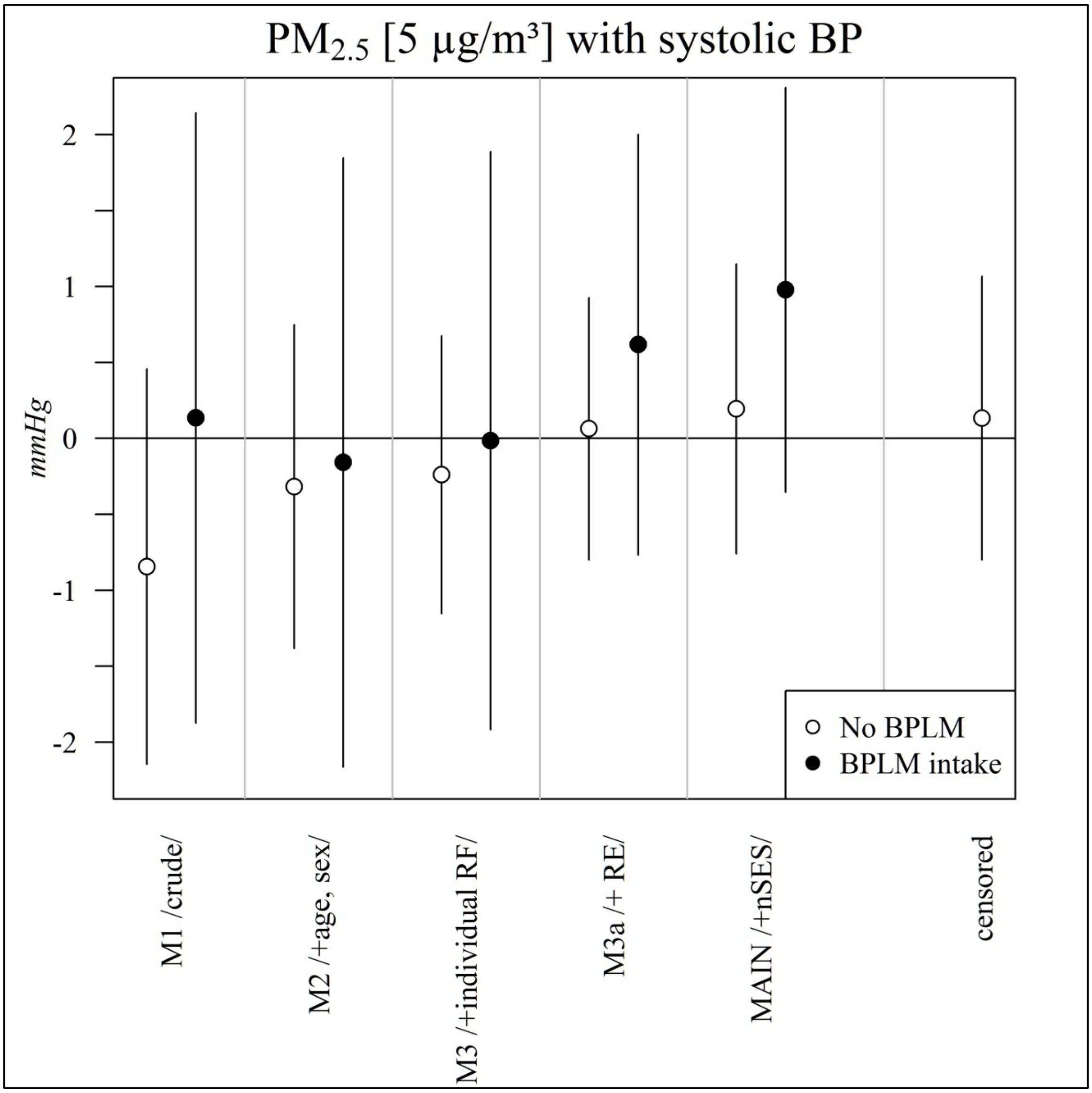

Figure S4. Association of $PM_{2.5}$ with systolic blood pressure: a comparison of adjustment models from crude to main in medicated and non-medicated with blood-pressure lowering medication, and censored regression. Legend: BP = blood pressure; BPLM = BP lowering medication; RF = risk factors; RE = random effect for neighborhood; nSES = neighborhood socio-economic status; change in mmHg with 95% confidence interval is presented.

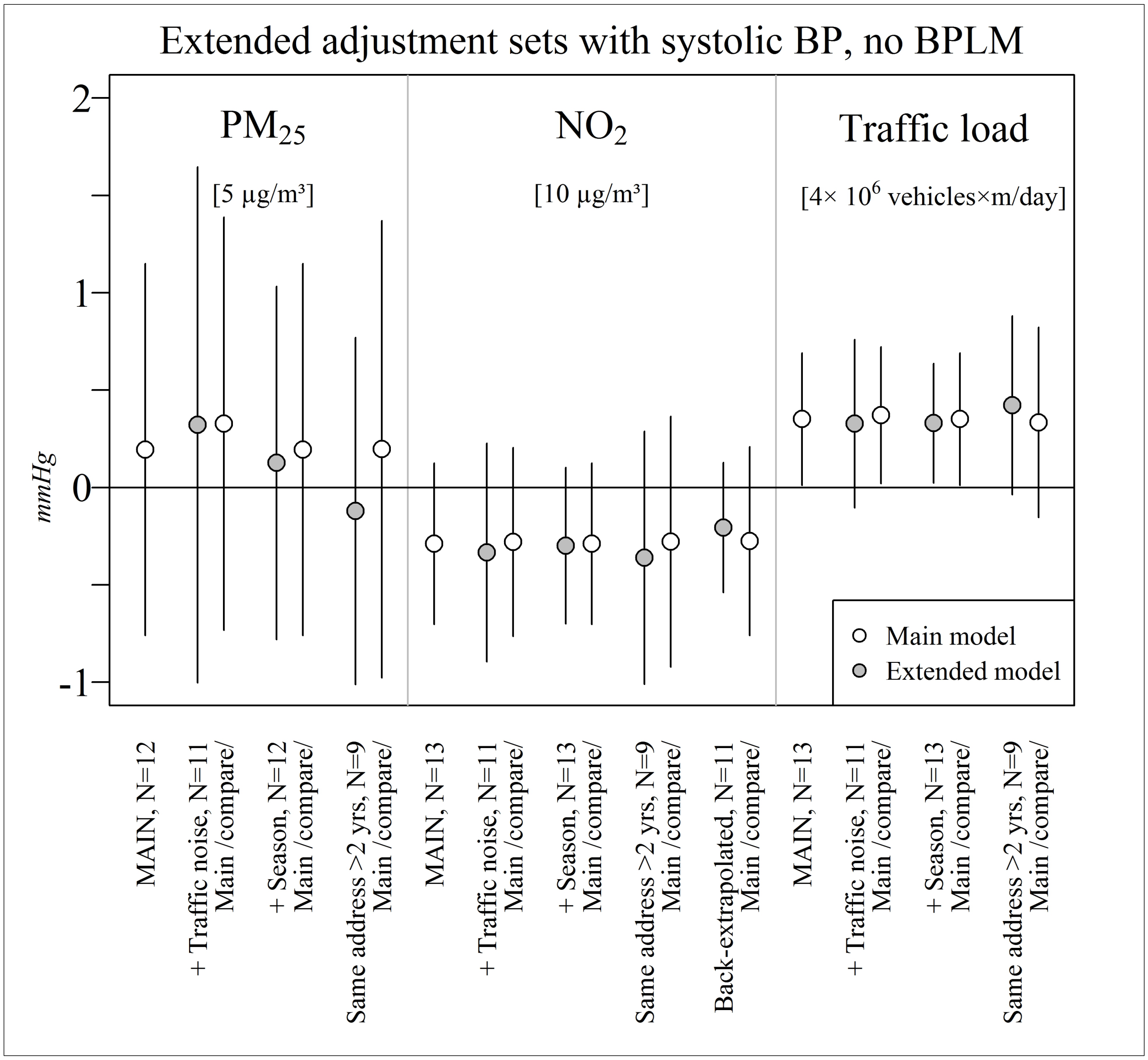

Figure S5. Extended adjustment sets and analysis with back-extrapolated exposures with systolic blood pressure in participants not medicated with blood pressure lowering medication. Legend: BP = blood pressure; BPLM = BP lowering medication; change in mmHg with 95% confidence interval is presented.

Main model: adjusted for age, sex, body mass index, smoking status, pack-years, passive smoking, alcohol consumption, physical activity, educational level, economic activity, neighborhood SES (including a random intercept for neighborhood). Cohorts included: FINRISK, HUBRO, 60-year-olds, SDPP, SNAC-K, TwinGene, EPIC-Umeå (only in analyses with NO_2 and traffic load), EPIC-MORGEN, EPIC-Prospect, HNR, KORA, SAPALDIA (Lugano site with $PM_{2.5}$, all 3 sites with NO_2 and traffic load), and REGICOR;

Main/compare/: Main model in the same cohorts and with the same participants as used for the respective sensitivity analysis;

Extended adjustment sets:

- + *noise:* adjusted for road traffic noise: SDPP and EPIC-Umeå excluded (data not available).
- + *season*: meteorological season included (same cohorts as in the main model).

Sensitivity analyses:

Same address > 2 yrs: we excluded participants who changed their residence during 2 years before BP measurement: KORA, EPIC-Prospect, EPIC-MORGEN excluded (data not available).

- Back-extrapolated NO_2: FINRISK, HUBRO excluded (back extratpolation not performed).

References

Ackermann-Liebrich U, Kuna-Dibbert B, Probst-Hensch NM, et al. 2005. Follow-up of the Swiss Cohort Study on Air Pollution and Lung Diseases in Adults (SAPALDIA 2) 1991–2003: methods and characterization of participants. Soz Praventivmed 50:245–263.

Beelen R, Hoek G, Vienneau D, Eeftens M, Dimakopoulou K, Pedeli X, et al. 2013. Development of NO2 and NOx land use regression models for estimating air pollution exposure in 36 study areas in Europe – the ESCAPE project. Atmos Environ 72:10–23.

Beulens JWJ, Monninkhof EM, Verschuren WMM, Schouw YT van der, Smit J, Ocke MC, et al. 2010. Cohort profile: the EPIC-NL study. Int J Epidemiol 39:1170–1178.

Cyrys J, Eeftens M, Heinrich J, Ampe C, Armengaud A, Beelen R, et al. 2012. Variation of NO2 and NOx concentrations between and within 36 European study areas: Results from the ESCAPE study. Atmos Environ 62:374–390.

Danesh J, Saracci R, Berglund G, Feskens E, Overvad K, Panico S, et al. 2007. EPIC-Heart: the cardiovascular component of a prospective study of nutritional, lifestyle and biological factors in 520,000 middle-aged participants from 10 European countries. European journal of epidemiology 22:129–141.

Eeftens M, Beelen R, Hoogh K de, Bellander T, Cesaroni G, Cirach M, et al. 2012a. Development of Land Use Regression models for PM(2.5), PM(2.5) absorbance, PM(10) and PM(coarse) in 20 European study areas; results of the ESCAPE project. Environ Sci Technol 46:11195–11205.

Eeftens M, Tsai M-Y, Ampe C, Anwander B, Beelen R, Bellander T, et al. 2012b. Spatial variation of PM2.5, PM10, PM2.5 absorbance and PMcoarse concentrations between and within 20 European study areas and the relationship with NO2 – Results of the ESCAPE project. Atmos Environ 62:303–317.

Eriksson A-K, Ekbom A, Granath F, Hilding A, Efendic S, Ostenson C-G. 2008. Psychological distress and risk of pre-diabetes and Type 2 diabetes in a prospective study of Swedish middle-aged men and women. Diabet Med 25:834–842.

European Commission. 2002. Directive 2002/49/EC of the European Parliament and of the Council of 25 June 2002 relating to the assessment and management of environmental noise. Official Journal of the European Union 2002. L189 of 18.7.2002, 12–25.

European Study of Cohorts for Air Pollution Effects (ESCAPE). 2012. Procedure for extrapolation back in time. Available: http://www.escapeproject.eu/manuals/Procedure_for_extrapolation_back_in_time.pdf [accessed 24 April 2014].

Grau M, Subirana I, Elosua R, Solanas P, Ramos R, Masiá R, et al. 2007. Trends in cardiovascular risk factor prevalence (1995-2000-2005) in northeastern Spain. Eur J Cardiovasc Prev Rehabil 14:653–659.

Holle R, Happich M, Löwel H, Wichmann HE. 2005. KORA–a research platform for population based health research. Gesundheitswesen 67 Suppl 1:S19–S25.

Jennrich RI, Robinson SM. 1969. A Newton-Raphson algorithm for maximum likelihood factor analysis. Psychometrika 34:111–123.

Lagergren M, Fratiglioni L, Hallberg IR, Berglund J, Elmståhl S, Hagberg B, et al. 2004. A longitudinal study integrating population, care and social services data. The Swedish National study on Aging and Care (SNAC). Aging Clin Exp Res 16: 158–168.

Magnusson PKE, Almqvist C, Rahman I, Ganna A, Viktorin A, Walum H, et al. 2013. The Swedish twin registry: establishment of a biobank and other recent developments. Twin Res Hum Genet 16:317–329.

Oudin A, Forsberg B, Strömgren M, Beelen R, Modig L. 2012. Impact of residential mobility on exposure assessment in longitudinal air pollution studies: a sensitivity analysis within the ESCAPE project. ScientificWorldJournal 2012:125818; doi:10.1100/2012/125818.

Peltonen M, Harald K, Männistö S,Männistö S, Saarikovski L, Peltomäki P, Lund L, et al.. 2008. The National FINRISK 2007 Study. Publications of the National Public Health Institute, Report No.: B34/2008 (article in Finnish). Available: http://www.julkari.fi/bitstream/handle/10024/78146/2008b34.pdf?sequence=1 [accessed 15 January 2014].

Riboli E, Hunt KJ, Slimani N, Ferrari P, Norat T, Fahey M, et al. 2002. European Prospective Investigation into Cancer and Nutrition (EPIC): study populations and data collection. Public Health Nutr 5:1113–1124.

Schmermund A, Möhlenkamp S, Stang A, Grönemeyer D, Seibel R, Hirche H, et al. 2002. Assessment of clinically silent atherosclerotic disease and established and novel risk factors for predicting myocardial infarction and cardiac death in healthy middle-aged participants: rationale and design of the Heinz Nixdorf RECALL Study. Risk Factors, Evaluation of Coronary Calcium and Lifestyle. Am Heart J 144:212–218.

Søgaard AJ, Selmer R, Bjertness E, Thelle D. 2004. The Oslo Health Study: The impact of self-selection in a large, population-based survey. Int J Equity Health 3:3.

Stang A, Moebus S, Möhlenkamp S, Dragano N, Schmermund A, Beck E-M, et al. 2006. Algorithms for converting random-zero to automated oscillometric blood pressure values, and vice versa. Am J Epidemiol 164:85–94.

Tjønneland A, Olsen A, Boll K, Stripp C, Christensen J, Engholm G, et al. 2007. Study design, exposure variables, and socioeconomic determinants of participation in Diet, Cancer and Health: a population-based prospective cohort study of 57,053 men and women in Denmark. Scand J Public Health 35:432–441.

Tobin MD, Sheehan NA, Scurrah KJ, Burton PR. 2005. Adjusting for treatment effects in studies of quantitative traits: Antihypertensive therapy and systolic blood pressure. Stat Med 24:2911–2935.

Wändell P-E, Wajngot A, Faire U de, Hellénius M-L. 2007. Increased prevalence of diabetes among immigrants from non-European countries in 60-year-old men and women in Sweden. Diabetes Metab 33:30–36; doi:10.1016/j.diabet.2006.07.001.

World Health Organization Collaborating Centre for Drug Statistics Methodology. Anatomical Therapeutic Chemical Classification Index with Defined Daily Doses 2006. Available from: http://www.whocc.no/atc_ddd_index/ [accessed 28 April 2014].
